# A Carboxy-terminal *Smarcb1* Point Mutation Induces Hydrocephalus Formation and Affects AP-1 and Neuronal Signalling Pathways in Mice

**DOI:** 10.1007/s10571-023-01361-5

**Published:** 2023-05-23

**Authors:** Aliska K. Brugmans, Carolin Walter, Natalia Moreno, Carolin Göbel, Dörthe Holdhof, Flavia W. de Faria, Marc Hotfilder, Daniela Jeising, Michael C. Frühwald, Boris V. Skryabin, Timofey S. Rozhdestvensky, Lydia Wachsmuth, Cornelius Faber, Martin Dugas, Julian Varghese, Ulrich Schüller, Thomas K. Albert, Kornelius Kerl

**Affiliations:** 1 Department of Paediatric Haematology and Oncology, University Children’s Hospital Münster, 48149 Münster, Germany; 2 Institute of Medical Informatics, University of Münster, 48149 Münster, Germany; 3 Department of Paediatric Haematology and Oncology, University Medical Center Hamburg-Eppendorf, 20251 Hamburg, Germany; 4 Research Institute Children’s Cancer Center, 20251 Hamburg, Germany; 5 Institute of Neuropathology, University Medical Center Hamburg-Eppendorf, 20251 Hamburg, Germany; 6 Swabian Children’s Cancer Center, Paediatrics and Adolescent Medicine, University Medical Center Augsburg, 86156 Augsburg, Germany; 7 Medical Faculty, Core Facility TRAnsgenic Animal and Genetic Engineering Models (TRAM), University of Münster, 48149 Münster, Germany; 8 Clinic of Radiology, Translational Research Imaging Center (TRIC), University of Münster, 48149 Münster, Germany; 9 Institute of Medical Informatics, Heidelberg University Hospital, 69120 Heidelberg, Germany

**Keywords:** SMARCB1 (BAF47), BAF complex, Brain development, Neurodevelopmental disorder, Hydrocephalus, Single-cell RNA sequencing

## Abstract

The BAF (BRG1/BRM-associated factor) chromatin remodelling complex is essential for the regulation of DNA accessibility and gene expression during neuronal differentiation. Mutations of its core subunit *SMARCB1* result in a broad spectrum of pathologies, including aggressive rhabdoid tumours or neurodevelopmental disorders. Other mouse models have addressed the influence of a homo- or heterozygous loss of *Smarcb1*, yet the impact of specific non-truncating mutations remains poorly understood. Here, we have established a new mouse model for the carboxy-terminal *Smarcb1* c.1148del point mutation, which leads to the synthesis of elongated SMARCB1 proteins. We have investigated its impact on brain development in mice using magnetic resonance imaging, histology, and single-cell RNA sequencing. During adolescence, *Smarcb1*^1148del/1148del^ mice demonstrated rather slow weight gain and frequently developed hydrocephalus including enlarged lateral ventricles. In embryonic and neonatal stages, mutant brains did not differ anatomically and histologically from wild-type controls. Single-cell RNA sequencing of brains from newborn mutant mice revealed that a complete brain including all cell types of a physiologic mouse brain is formed despite the SMARCB1 mutation. However, neuronal signalling appeared disturbed in newborn mice, since genes of the AP-1 transcription factor family and neurite outgrowth-related transcripts were downregulated. These findings support the important role of SMARCB1 in neurodevelopment and extend the knowledge of different *Smarcb1* mutations and their associated phenotypes.

**Supplementary Information:**

The online version contains supplementary material available at 10.1007/s10571-023-01361-5.

## Introduction

Neural development is accompanied by precisely coordinated changes in the chromatin state that determine the individual cells’ fate and function. Indispensable to this process is, among others, the BAF (BRG1/BRM-associated factor) complex, which is the mammalian counterpart of the yeast SWI/SNF chromatin remodelling complex. It modulates the chromatin architecture in an ATP-dependent manner to allow DNA accessibility and gene expression (Ronan et al. 2013). The BAF complex is composed of up to 15 subunits determining its time and tissue-specific function thus enabling the complex to regulate differentiation and stemness (Alfert et al. 2019). Its critical role is reflected by the fact that mutations of BAF subunits are often detected in neurodevelopmental disorders (Sokpor et al. 2017; Bögershausen and Wollnik 2018) and in > 20% of all human cancers (Kadoch and Crabtree 2015).

During neural development, the switch from the neural progenitor BAF (npBAF) to neuronal BAF (nBAF) complexes marks the transition from neural progenitor cells to post-mitotic, differentiating neurons. Both complexes are composed of one ATPase subunit, SMARCA4 (BRG1) or SMARCA2 (BRM), the core subunits SMARCB1, SMARCC1 and SMARCC2, and various other proteins, including ARID1A and ARID1B. However, the nBAF complex differs from the npBAF complex in the expression of (I) ACTL6B instead of ACTL6A, (II) DPF1 or DPF3 instead of PHF10 or DPF2 and (III) SS18L1 instead of SS18 (Lessard et al. 2007; Alfert et al. 2019).

The SMARCB1 subunit includes an amino(N)-terminal DNA-binding domain (DBD), two repeat motifs and a short coiled-coil region. The winged helix DBD initiates the recruitment of the BAF complex to target genes (Allen et al. 2015), while the carboxy(C)-terminal coiled-coil region is believed to interact with the nucleosome acidic patch (Valencia et al. 2019).

Mutations of *SMARCB1* are found in neurodevelopmental disorders as well as in a variety of malignancies (Holsten et al. 2018). Biallelic *SMARCB1* inactivation, mostly due to whole gene and exon deletions and truncating variants, is characteristic of the development of malignant rhabdoid tumours at a young age (Versteege et al. 1998; Holsten et al. 2018). Rhabdoid tumours are aggressive paediatric cancers that are usually localized in the central nervous system (atypical teratoid rhabdoid tumour, ATRT), the kidney (rhabdoid tumour of the kidney, RTK) or soft tissues (malignant rhabdoid tumour, MRT) (Nesvick et al. 2020; Nemes et al. 2022). By contrast, non-truncating variants of *SMARCB1*, which are mostly located in the exons one, two, eight and nine, are associated with late-onset tumours, such as schwannoma and meningioma (Holsten et al. 2018), and with neurodevelopmental disorders, such as intellectual disability with plexus hyperplasia (Kleefstra et al. 2012; Diets et al. 2019) and the Coffin–Siris syndrome (Santen et al. 2013; Tsurusaki et al. 2014). The latter is a rare congenital disorder characterised by developmental delay, microcephaly, coarse facial features, and aplastic or hypoplastic fifth finger/toenails (Coffin and Siris 1960; Tsurusaki et al. 2014). Thus, germline and somatic mutations of *SMARCB1* can lead to a wide range of pathologies. However, the functional causes are not yet fully understood.

While previously published mouse models have focused on the impact of homozygous (Moreno et al. 2014; Graf et al. 2022) or heterozygous (Filatova et al. 2019) *Smarcb1* loss on neuronal development and tumorigenesis, models for non-truncating and/or point mutations of *Smarcb1* are lacking. Yet, these are ultimately needed to decipher how the diverse array of mutant SMARCB1 leads to either uninhibited cell division in tumours or aberrant brain development in neurodevelopmental disorders.

In this study, we have established a new mouse model bearing the C-terminal *Smarcb1* c.1148del mutation. This mutation results in the synthesis of elongated SMARCB1 proteins, which in turn might alter the composition and function of the BAF complex. We characterized the development of mutant brains by a multi-faceted approach including magnetic resonance imaging (MRI), histology, and single-cell RNA sequencing (scRNA-seq).

## Methods

### Genetically Engineered Mouse Model

*Smarcb1*^*1148del*/+^ mice were generated by direct oocytes microinjections using the *CRISPR/Cas9* components together with the donor DNA oligo Smarb1c1148c (GGCGAATGAGGCGTCTTGCCAACACTGCCCAGCCTGGTGATGAAGACATCCATGCTCGAC, Eurofins Genomics) with subsequent embryo transfer. For the preparation of *CRISPR/Cas9* microinjection solution, commercially synthesized crRNA (Smarcb1CY_crRNA4) with the target sequence: GCCAACACTGCCCCAGCC, together with the tracrRNA and Alt-R™ S.p. Cas9 protein (Integrated DNA Technologies, #1081059) were mixed as follows: 200 pmol of crRNA were mixed with 200 pmol of tracrRNA in 10 mM potassium acetate and 3 mM HEPES (pH 7.5) buffer and incubated at 95 °C for 2 min, followed by cooling to room temperature. The annealed crRNA/tracrRNA complexes were mixed with Cas9 mRNA, Cas9 protein, and Smarb1c1148c template DNA oligo. The final concentrations of *CRISPR/Cas9* components in 0.6 mM HEPES (pH 7.5) and 2 mM potassium acetate microinjection buffer were as follows: crRNA (4 pmol/µL), tracrRNA (4 pmol/µL), Cas9 mRNA (10 ng/µL), Cas9 protein (25 ng/µL), Smarb1c1148c template DNA oligo (20 ng/µL). The final injection solution was filtered through millipore centrifugal columns and spun at 20000 g for 10 min at room temperature.

Microinjections were performed in B6D2F1 (hybrid between C57BL/6J and DBA strains) fertilized one-cell oocytes. Oocytes were removed from oviducts of superovulated B6D2F1 female mice in M2 medium (Behringer et al. 2014) supplemented with hyaluronidase (400 µg/mL, Sigma-Aldrich, #H3506), washed twice for removal of cumulus cells in M2 medium, transferred to KSOM medium (Behringer et al. 2014), and kept at 5% CO_2_ and 37 °C before injection. Cytoplasmic microinjections were performed in M2 medium using the transjector 5246 (Eppendorf), and Narishige NT-88NE micromanipulators attached to a Nikon Diaphot 300 inverted microscope. Oocytes that survived microinjections were transferred to oviducts of pseudopregnant CD1 foster mice and carried to term. Positively targeted F0 and F1 animals were identified by quantitative PCR (qPCR) and sequencing analysis of genomic DNA isolated from ear biopsies.

*Smarcb1*^*1148del*/+^ mice were maintained on the C57BL/6 background and were bred to obtain *Smarcb1*^*1148del*/1148del^ mice. Animals of both sexes were used for the experiments. Whole body weights of at least 3 mutant and 3 wild-type animals were measured daily between days 30 and 40. All mice were monitored regularly. Between 1 and 5 animals were housed per cage. Protocols and animal housing were in accordance with all guidelines provided by the local regulatory authorities (reference number TVA84-02.04.2015.A088 and TVA81-02.04.2018.A214; Government of NRW, Germany).

### Genotyping

DNA was extracted from ear biopsies by applying 200 µL lysis buffer (100 mM Tris, 5 mM EDTA, 0.2% (w/v) SDS, 200 mM NaCl), 7µL proteinase K (Roche, #03115828001) and 5µL ribonuclease A (20 mg/mL, Sigma-Aldrich, #R5000) and incubating overnight at 55 °C at 700 rpm. After enzyme inactivation at 85 °C for 5 min, the solution was centrifuged at 20800 g for 10 min at room temperature, DNA was precipitated using 2-propanol and washed with 70% (v/v) ethanol. After centrifugation (20800 g, 5 min) the DNA pellet was left to dry for at least 3 h before resuspension in nuclease-free water.

Subsequent genotyping was done by either qPCR or sequencing. RT-qPCR was performed using 10 µL Luna Universal Probe qPCR Master Mix (New England BioLabs, #M3004), 8 pmol of each forward primer Fw (5′-CCCTACTTCAGGCGAATG AG-3′, Eurofins Genomics) and reverse primer Rv (5′-GGTCGAGCATGGATGTCTTC-3′, Eurofins Genomics) and 20 ng DNA in a total volume of 20 µL per single reaction. Additionally, 0.4 µL qPCR Probe (Affinity Plus® Mini Probe 5′ 6-FAM™ / 3′ IB®FQ, IDT®) were included, detecting either the wild-type allele (5′-/56-FAM/TGC+CC+CA+GCCT/3IABkFQ/-3′) or the mutant allele (5′-/56-FAM/TG+CC+CA+GCCT/3IABkFQ/-3′). The RT-qPCR was run on a C1000 touch thermal cycler with a CFX96 optical reaction module (Bio-Rad Laboratories) with the following protocol: 95 °C for 1 min, followed by 50 cycles of 95 °C for 15 s, 57 °C for 30 s and 40 °C for 10 s. Analysis was conducted with the Bio-Rad CFX Maestro 1.1 software.

For sequencing, a PCR was performed using 7 µL GoTaq® G2 Master Mix (Promega, #M7423), 7 pmol of each forward primer Fw (5′-GGCCCCAGGGTACATTTTCTC-3′) and reverse primer Rv (5′-GGGACAGTGTTGGGGTTTAGC-3′) (Eurofins Genomics) in a total volume of 10 µL, then running the following programme at a Mastercycler nexus GSX1 (Eppendorf): 95 °C for 3 min, 40 cycles of 95 °C for 30 s, 60 °C for 45 s, 72 °C for 1 min, followed by a final extension at 72 °C for 5 min. A 1.5% (w/v) agarose gel (Roth, #2267.5) containing SYBR Safe (Invitrogen, #S33102) was run at 100 V for 30 min. The DNA was extracted using the QIAquick® Gel Extraction Kit (QIAGEN, #28704). Eluted DNA was sequenced by Eurofins Genomics (Mix2Seq Kit, Eurofins Genomics, #3094-0ONMSK) using primer Fw.

### Murine Primary Cells

Isolated brains from *Smarcb1*^1148del/1148del^ and *Smarcb1*^+/+^ P0 mice were divided into a supratentorial and an infratentorial part and each was minced with scalpels on ice. For enzymatic dissociation, 2–3 mL of a solution containing 1vial papain (Worthington, #LK003178), 5 mL pre-equilibrated DMEM:F12 (Gibco, #11330-032) and 340 µg DNAse I (Worthington, #2139) diluted in 250 µL DMEM:F12 were added to each sample, prior to incubation at 37 °C in 5%CO_2_ for 30 min. The samples were gently shaken every 3–5 min and finally transferred with 1 mL wide bore tips (Starlab, #E1011-9000) onto a 40 μm cell strainer (Corning, #352340). The reaction was stopped with 2 mL PBS/10%BSA, followed by washing with 10 mL PBS. The samples were centrifuged at 1200 rpm for 8 min at room temperature. The pellet was then resuspended in neural stem cell (NSC) medium, containing DMEM:F12 (Gibco, #11330-032), 100 U/mL penicillin–streptomycin (Gibco, #15140-122), 1 × B-27™ supplement minus vitamin A (Thermo Fisher Scientific, #12587-010), 20 ng/mL recombinant murine EGF (Peprotech, #315-09) and 20 ng/mL recombinant murine FGF-basic (Peprotech, #450-33), for a subsequent scRNA-seq procedure or cell culture.

### Cell Culture

Isolated brain cells were cultured in uncoated wells in NSC medium. NIH3T3 cells (ACC 59, DSMZ, Braunschweig, Germany) were cultured in uncoated wells in Dulbecco’s modified Eagle medium (DMEM, Gibco, #41966-029) with the addition of 10% FBS (Sigma-Aldrich, #F7524). For passaging, both murine brain cells and NIH3T3 cells were detached with 0.05% trypsin (Gibco, #25300054), incubating for 1–2 min at 37 °C. *Smarcb1*-negative T15 cells from a murine peripheral T-cell lymphoma, not otherwise specified, (gift from Charles W. M. Roberts, Dana–Farber Cancer Institute, BOS, USA) were cultured in RPMI-1640 Medium (Sigma-Aldrich, #8758) containing 100 U/mL penicillin–streptomycin (Gibco, #15140-122), 2 mM L-Glutamine, 10 mM HEPES (Gibco, #15630080), 1 mM sodium pyruvate (gibco, #11360070), 50 µM 2-mercaptoethanol (Gibco, #21985-023) and FBS (Sigma-Aldrich, #F7524). All cells were maintained at 37 °C and 5% CO_2_ and were regularly tested for mycoplasma contamination.

### Immunoblotting

Whole-cell lysates from cultured cells were obtained by resuspending the cell pellets in 100 µL of PSB (phosphorylation solubilization buffer: 0.5% NP40/IGEPAL (Sigma-Aldrich, #I8896), 50 mM HEPES (AppliChem, #A1069), 100 mM NaF (Roth, #P756), 10 mM Na_4_P_2_O_7_ (Sigma-Aldrich, #71515), 2mM Na_3_VO_4_ (AppliChem, #A2196), mM EDTA (AppliChem, #A2937), 2 mM Na_2_MoO_4_·2H_2_O (AppliChem, #A2193) containing cOmplete Mini protease inhibitor cocktail tablets (1 tbl/10 mL PSB, Roche, #11697498001)). The pH was adjusted to 7.35 with 5 M NaOH. Whole-cell lysates from isolated organs were obtained by adding 1mL RIPA buffer (50 mM Tris-HCl, 150 mM NaCl (Roth, #3957), 1% NP-40/IGEPAL (Sigma-Aldrich, #I8896), 0.5% sodium deoxycholate (AppliChem, #A1531), 0.1% (w/v) SDS (AppliChem, #A2572) supplemented with cOmplete Mini protease inhibitor cocktail tablets (1 tbl/10 mL RIPA buffer, Roche, #11697498001) to each organ in a 1.5 mL capsule. The capsules were snap-frozen in liquid nitrogen and shaken in a Mikro-Dismembranator U (Braun Biotech) at 2000 rpm for 4 min. Soluble lysates were recovered by centrifugation at 14000 × *g* for 20 min at 4 °C.

Subsequently, protein concentrations were determined colourimetrically by a BCA protein assay (Pierce, #23227) at a BioPhotometer Plus (Eppendorf, #6132). SDS-polyacrylamide gel electrophoresis was performed in 12.5% acrylamide gels (Bio-Rad, #1610156) with 20 µg protein each for cell culture and spleen lysates and with 30 µg protein each for brain, lung, liver and kidney lysates. For this, the lysates were supplemented with loading buffer and reducing agent [1:5 v/v from a 125 mM TrisHcl, 2.5% SDS (AppliChem, #A2572), 40% glycerol (Sigma, #G-5516), bromophenol blue (AppliChem, #A3640), 4% ß-mercaptoethanol solution (Sigma-Aldrich, #M6250)] and heated at 98 °C for 10 min. Blotting was carried out with the Trans-Blot® Turbo™ System (BioRad, #1704150) and nitrocellulose membranes (Bio-Rad, #1620115) using the “STANDARD SD, 2 mini gels” programme.

**Fig. 1:**
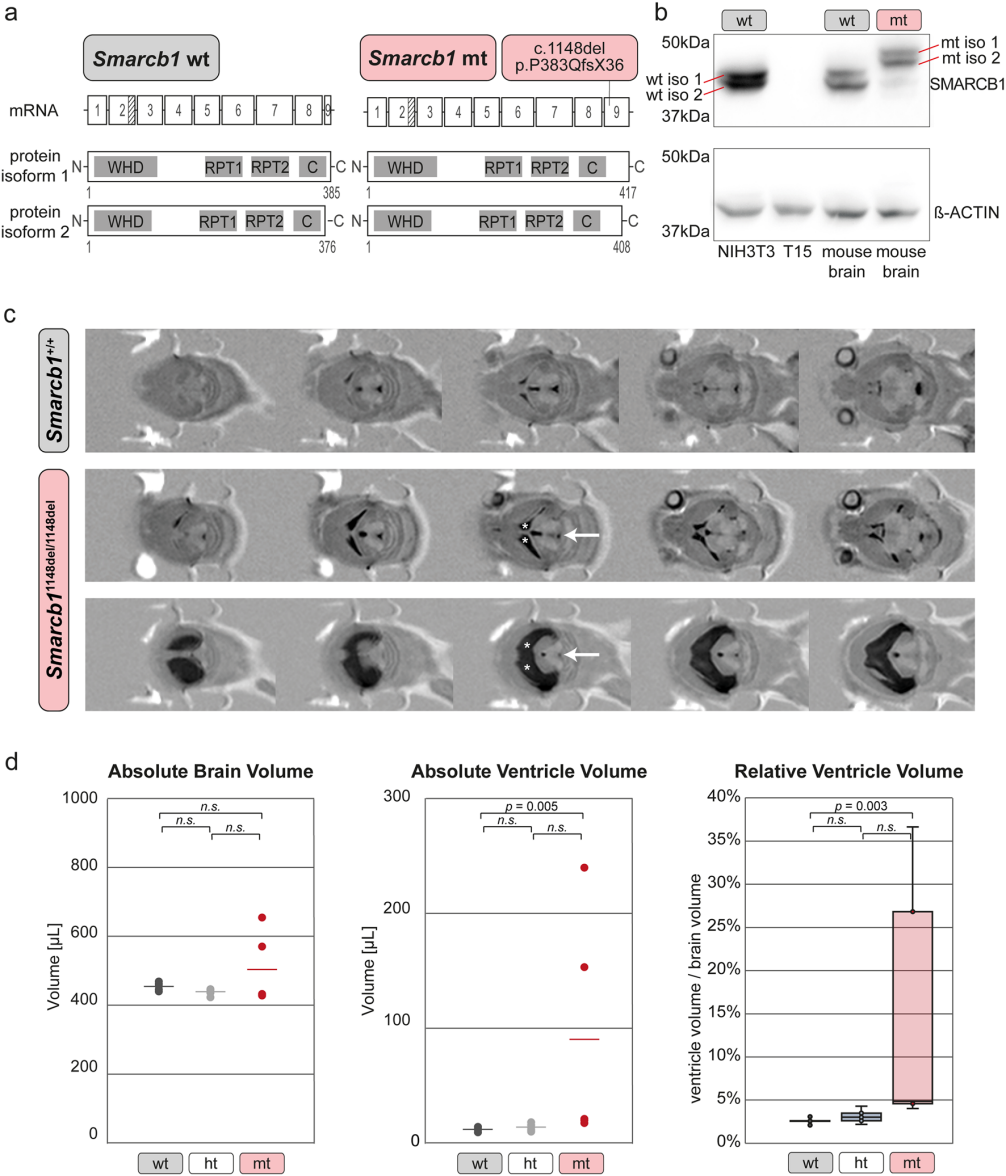
Enlarged cerebral ventricles in a *Smarcb1* c.1148del mutant mouse model. **a** Scheme depicting the structure of murine *Smarcb1* wild-type (wt) mRNA (top left) and the domain structure of both isoforms of the SMARCB1 wt protein (bottom left). Scheme showing the location of the c.1148del mutation in *Smarcb1* mutant (mt) mRNA (top right) and the resulting elongated SMARCB1 mt isoforms (bottom right). Striped areas indicate segments at the 3′ end of exon 2 that are present in isoform 1 and missing in isoform 2 due to an alternate in-frame splice junction. **b** Immunoblot showing different isoforms of wild-type (wt iso 1/2) and mutant (mt iso 1/2) SMARCB1 proteins in whole-cell lysates of brain cells isolated from newborn *Smarcb1*^+/+^ (wt) and *Smarcb1*^1148del/1148del^ (mt) mice. Lysates from the mouse embryonic fibroblast cell line NIH3T3 and a *SMARCB1*-negative peripheral T cell lymphoma cell line (T15) were included as positive and negative controls, respectively, and beta-actin antibody was used as a loading control on the same immunoblot. **c** MRI of wild-type and mutant *Smarcb1*^1148del/1148del^ mice mouse brains. The brains of mutant mice show different degrees of enlarged lateral ventricles (asterisks) and smaller aqueducts (arrows). **d** Graphs showing comparisons of absolute brain volumes and absolute or relative ventricular volumes in age-matched (P35–P40) *Smarcb1*^+/+^ mice (wt) (*n* = 5: 4 male, 1 female), *Smarcb1*^1148del/+^ (ht) (*n* = 5: 4 male, 1 female) and *Smarcb1*^1148del/1148del^ (mt) (*n* = 5: 3 male, 2 female) mice. Manual quantifications were performed using ImageJ, and measurements were subjected to a Kruskal–Wallis test. *WHD* winged helix DNA binding, *RPT1* repeat 1, *RPT2* repeat 2, *CC* coiled-coil domain

Afterwards, membranes were blocked with 5% milk (powdered milk (Carl Roth, #T145.2) dissolved in TBS-Tween). Incubation with primary antibodies (for blots with cell culture, liver and kidney lysates: Mouse Anti-BAF47, BD transduction laboratories, #612110, 1:1000; for blots with brain, lung and spleen lysates: Rabbit Anti-SMARCB1, Sigma-Aldrich, #HPA018248, 1:200; for all blots: Mouse Anti-ß-Actin, Santa Cruz Biotechnology, #SC-47,778, 1:10000) was conducted overnight at 4 °C. All antibodies have been successfully used and tested for antigen specificity before (Carugo et al. 2019; Zou et al. 2021). Complementary, the specificity of the Mouse Anti-BAF47 and the Rabbit Anti-SMARCB1 antibodies was validated by using it on the *Smarcb1*-wild-type cell line NIH3T3 and the *Smarcb1*-negative cell line T15 (Fig. 1b, Online Resource 4a).

Incubation with secondary antibodies (Peroxidase-conjugated Goat anti-mouse, Jackson Immuno Research, Hamburg, Germany, #115-035-044, 1:5000; Peroxidase-conjugated Goat anti-rabbit, Jackson Immuno Research, #111-035-045; 1:10000) was carried out for 1 h at room temperature. Chemiluminescence detection was realized with Western Lightning® ECL Pro Solution (PerkinElmer, #NEL120001EA) and a FUSION-SL Advanced Imager (Vilber).

### Magnetic Resonance Imaging

In vivo MRI was conducted on an Achieva 3.0 T Philips MR System operating at 127.74 MHz using a dedicated mouse radio frequency coil (Philips Research Laboratories 3T Solenoid Coil). Mice were narcotized with inhaled isoflurane (initiation 4% and preservation 1.2%, Abbott, #213111). 17 contiguous transversal slices with a slice thickness of 0.5 mm were acquired with a T1IR sequence and reconstructed to 0.15 mm in-plane pixel resolution. The T1-weighed images were analysed with ImageJ (Version 1.53q). Brain and ventricle volumes were determined from the measured brain or ventricle area multiplied by the slice thickness. Data processing and statistical tests were performed with Microsoft Excel 2016 and IBM SPSS Statistics (Version 28.0.1.1(14)).

### Immunohistochemistry

For histological evaluation, mouse brains (week 4–12) or entire heads of embryos/newborn pups (E13.5/P0) were fixed in 4% PFA for at least 24 h. Afterwards, they were dehydrated, embedded in paraffin and 4 μm sections were cut. Hematoxylin and eosin (H&E) stainings were performed according to standard protocols. For immunohistochemical stainings, the ultraView DAB Detection Kit was used on a Ventana Benchmark xt system (both Roche Diagnostics) according to the manufacturers’ instructions. The following antibody was used: anti-SMARCB1 (Clone 25/BAF47, BD Biosciences, #612110, 1:50) (Bruder et al. 1999; Carugo et al. 2019). For adolescence and P0, brain sections from at least three mutant animals were analysed. For E13.5, brain sections of two mutant animals were stained.

### Single-Cell RNA Sequencing of Mouse Brains

Single-cell suspensions obtained from the supratentorial and infratentorial parts of two S*marcb1*^1148del/1148del^ and two *Smarcb1*^+/+^ P0 mice were stained with 7-Aminoactinomycin D (7-AAD) (eBioscience™, #00-6993-50). Non-viable, 7-AAD-positive cells were removed by fluorescence-activated cell sorting (BD FACS Aria II, BD FACS Diva Software), followed by manual counting with trypan blue staining (Sigma-Aldrich, #T8154). Approximately 25,000 single cells of each sample were processed for scRNA-seq using the Chromium Next GEM Single Cell 3′ GEM, Library & Gel Bead Kit v3.1 (10X Genomics, #1000121), the Chromium Next GEM Chip G (10X Genomics, #1000120) and the Single Index Kit T set A (10X Genomics, #1000213) according to the manufacturer’s instructions. In brief, single-cell GEM (Gel Beads-in-Emulsion) were generated by the Chromium Controller, followed by GEM-RT (reverse transcription), Dynabeads cleanup, cDNA amplification, SPRIselect beads cleanup and Library Construction. Quality, purity, size and concentrations of cDNA and libraries were measured by a tapestation 2200 (Agilent Technologies). All libraries were sequenced separately by the NextSeq 500 sequencing platform (v 2.5 chemistry, 75 cycle kit) at the Core Facility Genomics (Medical Faculty of the Westfälische Wilhelms-Universität Münster, Münster).

### Preprocessing of Single-Cell RNA Sequencing Data

Eight murine scRNA-seq samples were processed with the 10X Genomics CellRanger v3.0.2 program suite (Zheng et al. 2017). First, the raw 10 × scRNA-seq data was converted to the standard fastq format with CellRanger’s mkfastq function. The CellRanger count algorithm was subsequently used to align the sample data against the murine reference transcriptome mm10 v3.0.0 with default values, and the resulting filtered feature matrices were checked for basic quality metrics with the CellRanger web summary reports. Samples with a sequencing saturation of < 35% were re-sequenced.

### Analysis of Single-Cell RNA Sequencing Data

Further analyses of the CellRanger data were conducted with R v4.0.5 (RCoreTeam21 2021) and Seurat v4.0.5 (Hao et al. 2021). For all samples, a minimum of 3 cells and a minimum feature number per cell of 200 were required; all cells with more than 25% of mitochondrial genes were removed from the analysis. Furthermore, outlier cells with a high nCount_RNA value were classified as doublets and removed from the analysis; depending on the sample’s cell distribution, the threshold for filtering was adapted (30,000 to 50,000, mean = 37,500). The filtered sample data was transformed with Seurat’s SCTransform function and default parameters, and a principal component analysis (PCA) was used for dimension reduction. Subsequently, integration anchors were identified based on the normalization method SCT and RPCA as reduction. The dataset was then integrated and clustered with a resolution parameter of 0.5. Uniform manifold approximation and projections (UMAP) and Seurat feature plots were chosen to visualize both the sample distribution and a set of predefined features and gene signatures. Additionally, Seurat’s FindMarkers function was used to compute differentially expressed marker genes for each cluster. Based on these calculated gene lists and marker plots, murine cell types were manually assigned to the Seurat clusters. The transcriptomic single-cell atlas of the Developing Mouse Brain (La Manno et al. 2021; www.mousebrain.org/development) and complementary publications for embryonic and perinatal brain development (Carter et al. 2018; Loo et al. 2019) were used as a reference for cell type annotation. Subclusterings were performed for all clusters with inhomogeneous marker plot results, so that the final clustering consisted of 37 distinct clusters.

As an additional layer of information, eight superclusters were introduced to structure the identified Seurat clusters further, and the resulting distribution of cells was analysed separately for the two murine groups and visualized as pie charts and bar charts with Microsoft Excel 2016. Gene Ontology term analysis was performed using ToppGene Suite (Chen et al. 2009; https://toppgene.cchmc.org/). Furthermore, expression values for chosen marker genes were extracted for both mouse groups and visualized with ggplot’s boxplot function (Wickham 2016). Heatmaps were used to visualize the expression of chosen marker genes in different cluster sets (https://cran.r-project.org/package=pheatmap). For the full murine data, a marker list published by Tirosh et al. (2016) was then used as input for Seurat’s CellCycleScoring function to identify cell cycle phases in the integrated dataset. Additional data exploration was performed with the R/Bioconductor package iSEE v2.6.0 (Rue-Albrecht et al. 2018).

### Mutational Spectrum of *SMARCB1*

For published *SMARCB1* mutations found in somatic cancers, we referred to the COSMIC cancer database, which reported nearly 2000 cases of *SMARCB1* mutations in tumour cells and 31 cases of the *SMARCB1* c.1148del/p.P383RfsX100 mutation (Tate et al. 2019; https://cancer.sanger.ac.uk/cosmic). Additional PubMed-based investigation included the following terms: “BAF”, “SWI/SNF”, “mutation”, “SMARCB1”, “BAF47”, “INI1”, “Coffin–Siris Syndrome”, “CSS”, “SWI/SNF-related intellectual disability disorders”, “SSRIDD”, “intellectual disability”, “Hydrocephalus”, “neurodevelopmental disorder”. The variants, summarized in Online Resource 2, were visualized with the R/Bioconductor package trackViewer’s lollipop function (Ou and Zhu 2019) and Adobe Illustrator 2022. For clarity, only cancer mutations with a reported count ≥ 5 and all neurodevelopmental disorder mutations are displayed in Online Resource 1.

### Statistics

A sample size of *n* ≥ 3 was chosen for all statistical analyses, with the single-cell analyses of two animals (four samples) per genotype being an exception. The allocation of the animals to the statistical groups was conducted according to their genotype. The evaluation was carried out without blinding the experimenter. Statistical tests were performed with IBM SPSS statistics (Version 28.0.1.1(14)) and R v4.0.5 (RCoreTeam21 2021). Data were tested for normal distribution using the Kolmogorov-Smirnov test and the Shapiro-Wilk test or the Anderson–Darling test for single-cell data. For comparisons of two groups, for normally distributed data, a two-tailed unpaired *t*-test was applied, and for the remaining data, a two-sided unpaired Mann-Whitney-*U*-Test was performed. When comparing more than two groups that were not normally distributed, the Kruskal-Wallis test was used instead. A subsequent Bonferroni correction was used to adjust for multiple testing. For detailed information on statistical tests, see the statistical report in the supplementary material.

Figures were created using Microsoft Excel 2016 and Adobe Illustrator 2022.

## Results

### Establishment of a Genetically Engineered Mouse Model with a *Smarcb1 *c.1148del Mutation

A comprehensive study of various databases and individual publications confirmed a complex spectrum of *SMARCB1* mutations (Online Resources 1, 2) (Tsurusaki et al. 2012, 2014; Kleefstra et al. 2012; Santen et al. 2013; Wieczorek et al. 2013; Gossai et al. 2015; Tate et al. 2019; Diets et al. 2019; Filatova et al. 2019; Sekiguchi et al. 2019; Cheng et al. 2021). These include approximately 900 different somatic cancer mutations (Tate et al. 2019; COSMIC Cancer database, https://cancer.sanger.ac.uk/cosmic), including whole gene deletions and point mutations such as *SMARCB1* c.1148del/p.P383RfsX100 (COSM1057), as well as neurodevelopmental disorders such as intellectual disability with plexus hyperplasia and the Coffin–Siris syndrome.

To obtain insight into the functional consequences of a C-terminal, non-truncating mutation, we established a genetically engineered mouse model. These mice carry a cytosine point deletion in exon nine, resulting in a frameshift of 36 amino acids until the following stop codon (*Smarcb1* c.1148del/p.P383QfsX36). The resulting protein contains 32 additional amino acids located C-terminal to the coiled-coil region (Online Resource 3a), which, according to computational prediction tools (Gasteiger et al. 2005, https://web.expasy.org/compute_pi/), increases its molecular weight by 3.4 kDa. Due to alternative splicing within exon two of the *Smarcb1* pre-mRNA, the wild-type (wt) SMARCB1 protein is expressed in two isoforms of 385 and 376 amino acids in length (wt iso 1 and wt iso 2, respectively) (Fig. 1a, b) (Bruder et al. 1999). Similarly, the *Smarcb1* c.1148del pre-mRNA undergoes alternative splicing and encodes the mutant (mt) SMARCB1 isoforms with a length of 417 and 408 amino acids (mt iso 1 and mt iso 2), respectively (Fig. 1a, b).

In immunoblots, we detected roughly equal expression levels for wt iso 1 and 2 SMARCB1 proteins in NIH3T3 embryonic fibroblasts (Fig. 1b, Online Resource 4a). The same observation was made when using whole-cell lysates from brain cells of newborn wild-type mice, while mt iso 1 and 2 SMARCB1 proteins were exclusively detected in corresponding lysates from *Smarcb1*^1148del/1148del^ mice (Fig. 1b, Online Resource 4a). Further immunoblots demonstrated the expression of all isoforms in organs of adult wild-type and *Smarcb1* mutant mice (Online Resource 3b, 4b,c,d). Heterozygous mice expressed both wt and mt SMARCB1 isoforms, yet, the *Smarcb1* c.1148 mutation does not appear to induce preferential expression of either isoform. One exception can be identified in liver cells, where only isoform 1 was detected in all three genotypes (Online Resource 3b, 4d).

Taken together, the human *SMARCB1* c.1148delC mutation has been transferred into a mouse model. It leads to the expression of prolonged SMARCB1 proteins in all organs examined, including the brain.

### *Smarcb1*^1148del/1148del^ Mutation Impairs Brain Development and Growth

Heterozygous *Smarcb1*^1148del/+^ mice were crossed to obtain homozygous *Smarcb1*^1148del/1148del^ mice, which were born at expected Mendelian ratios. Newborn *Smarcb1*^1148del/1148del^ mice were viable and did not show any apparent anomalies at the time of birth.

To visualize brain structures and ventricle shape, MRI was performed on five *Smarcb1*^+/+^ mice, five *Smarcb1*^1148del/+^ mice and five *Smarcb1*^1148del/1148del^ mice, each aged between 35 and 40 days. Images showed enlarged lateral ventricles filled with cerebrospinal fluid (CSF) in all *Smarcb1*^1148del/1148del^ mice, while their third ventricles appeared barely dilated. In addition, the images of *Smarcb1*^1148del/1148del^ brains suggested an aqueduct stenosis (a known cause of hydrocephalus), while other brain structures such as the thalamus and cerebellum appeared largely unaltered (Fig. 1c, Online Resource 5). The lateral ventricles of the heterozygous *Smarcb1*^1148del/+^ mice appear minimally enlarged, but beyond that, the brains show no other abnormalities (Online Resource 5).

Quantifications of the brain and ventricle volumes revealed similarly sized brains in *Smarcb1*^1148del/1148del^ (wt), *Smarcb1*^1148del/+^ (ht) and *Smarcb1*^1148del/1148del^ (mt) mice (absolute brain volume wt: *M* = 456.1 µL, *SD* = 11.8; ht: *M* = 440.8 µL, *SD* = 9.8; mt: *M* = 505.2 µL, *SD* = 104.3; *z* = 2.06, *p* = 0.357). In striking contrast, ventricle size was increased significantly in *Smarcb1*^1148del/1148del^ animals compared to *Smarcb1*^+/+^ animals (absolute ventricle volume wt: *M* = 11.7 µL, *SD* = 1.7; mt: *M* = 90.4 µL, *SD* = 102.1, *z* = -8.0, *p* = 0.005 | relative ventricle volume wt : *M* = 2.6%, *SD* = 0.3 ; mt : *M* = 15.4%, *SD* = 15.3 ; *z* = -8.4, *p* = 0.003). The relative ventricle volumes ranged between 2.1% and 3.1% in *Smarcb1*^+/+^ mice compared to 4.0% and 36.7% in *Smarcb1*^1148del/1148del^ mice. The absolute and relative ventricle volumes of heterozygous *Smarcb1*^1148del/+^ animals (absolute ventricle volume ht: *M* = 13.63 µL, *SD* = 3.3 | relative ventricle volume ht: *M* = 3.1%, *SD* = 0.8) did not differ significantly from those of *Smarcb1*^1148del/1148del^ and *Smarcb1*^+/+^ animals (Fig. 1d).

Moreover, 60% of *Smarcb1*^1148del/1148del^ mice developed externally visible hydrocephalus within the first 50 days of life. Of 15 *Smarcb1*^1148del/1148del^ mice and 15 *Smarcb1*^+/+^ mice observed postnatally over a period of 50 days, 9 *Smarcb1*^1148del/1148del^ mice and 0 *Smarcb1*^+/+^ mice showed externally visible hydrocephalus (average observational time point: 32 days [15; 47 days]) (Online Resource 3c).

Furthermore, *Smarcb1*^1148del/1148del^ mice showed delayed gain of weight: *Smarcb1*^1148del/1148del^ mice were 35% lighter on day 30 (wt: *M* = 16.0 g, *SD* = 1.7; mt: *M* = 10.4 g, *SD* = 2.5; *t*(16) = 5.60, *p* < 0.001), 38% lighter on day 35 (wt: *M* = 18.9 g, *SD* = 2.2; mt: *M* = 11.8 g, *SD* = 1.5, *t*(13) = 6.5, *p* < 0.001) and 21% lighter on day 40 (wt: *M* = 21.0 g, *SD* = 2.6; mt: *M* = 15.8 g, *SD* = 2.4; *t*(7) = 3.13, *p* = 0.016) (Online Resource 3d).

**Fig. 2:**
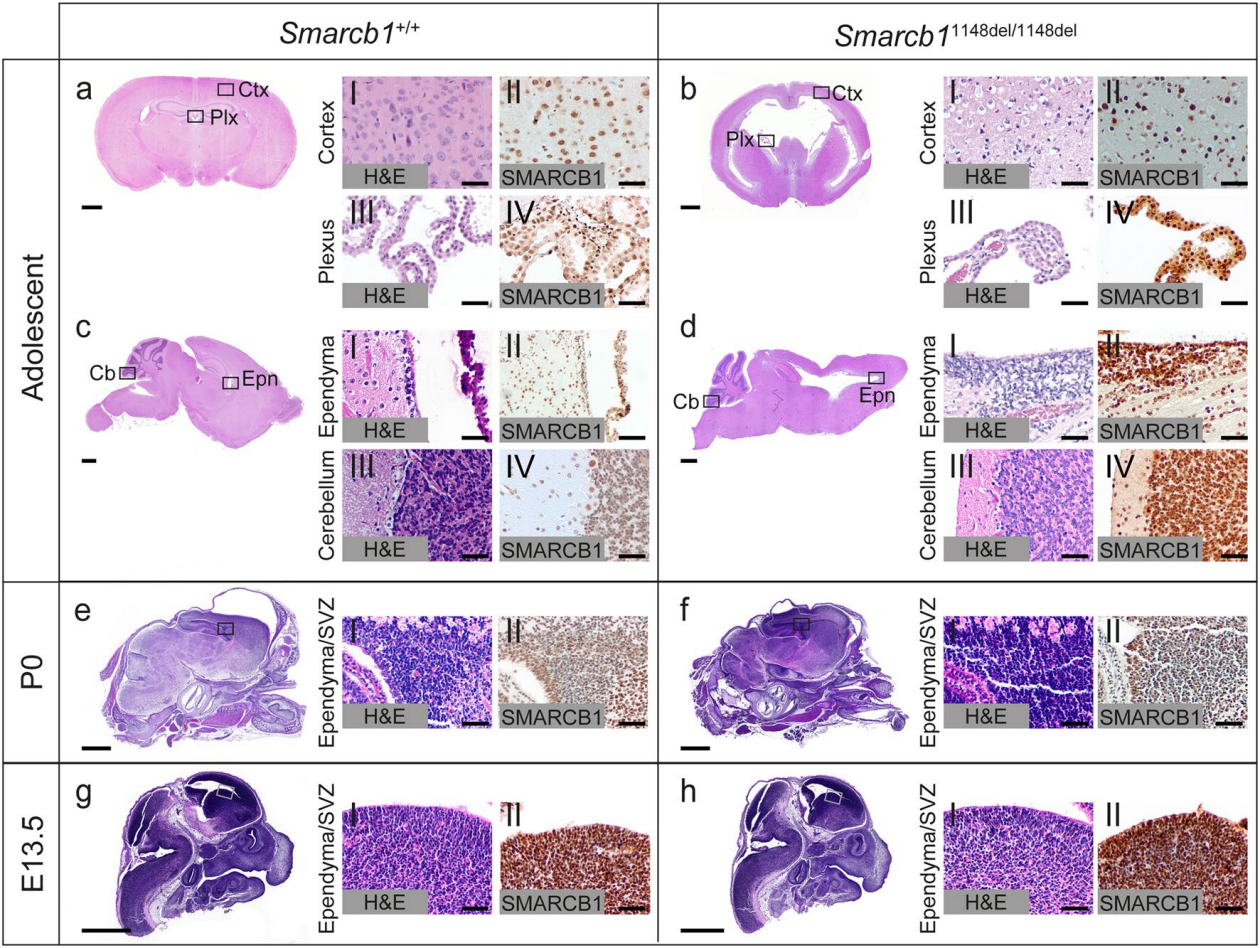
Histological characterization of the *Smarcb1* c.1148Cdel mouse model. **a, b, c, d** Hematoxylin and Eosin (H&E) (I and III) and anti-SMARCB1 (II and IV) antibody stainings of brain sections from adolescent (week 4–8) *Smarcb1* wild-type (**a**, **c**) and c.1148del (**b**, **d**) mutant mice. High-power images of the cortex (Ctx), the choroid plexus (Plx), the ependyma (Epn) and the cerebellum (Cb) are shown. *n* ≥ 3 animals. **e, f** H&E (I) and anti-SMARCB1 (II) antibody stainings of brain sections from newborn (P0, day of birth) *Smarcb1* wild-type (**e**) and c.1148del (**f**) mutant mice. High-power images of the ependyma including the subventricular zone (SVZ) are shown. *n* ≥ 3 animals. **g, h** HE (I) and anti-SMARCB1 (II) antibody stainings of brain sections from embryonal (E13.5) *Smarcb1* wild-type (**g**) and c.1148del (**h**) mutant mice. High-power images of the ependyma including the SVZ are shown. *n* = 2 animals. Scale bar: 1000 μm in full brain images and 100 μm in high power images (20x magnification)

Next, we investigated the impact of the *Smarcb1* c.1148del mutation on a histological level. We performed H&E and anti-SMARCB1 immunohistochemical (IHC) stainings on brain sections from adolescent (week 4–8), newborn (P0) and embryonal (E13.5) *Smarcb1*^+/+^ and mutant *Smarcb1*^1148del/1148del^ mice (Fig. 2a–h). This analysis revealed that mutant adolescent mice have enlarged lateral ventricles and a thinned cortex (Fig. 2a–d). Whether the latter is the cause or consequence of the hydrocephalus remains unclear. Other brain regions like the choroid plexus, the ependyma incl. the subventricular zone, and the cerebellum show no abnormalities (Fig. 2b-III, d-I, d-III). Hyperproliferation of the choroid plexus as a cause of hydrocephalus can thus be largely excluded. No differences to wild-type animals were observed at the earlier time points P0 and E13.5 (Fig. 2e–h). In general, all cells throughout the brain are SMARCB1-positive (Fig. 2b-II, b-IV, d-II, d-IV, f-II, h-II). Areas of tumour formation or cellular hyperproliferation, as found in *Smarcb1*-negative ATRT models, are not detectable (Graf et al. 2022) (Fig. 2b, d, f, h). In addition, the brains of heterozygous *Smarcb1*^1148del/+^ mice were examined at different time points, but showed no abnormalities compared to wild-type mice (Online Resource 6).

In conclusion, *Smarcb1*^1148del/1148del^ mice are 10–30% lighter compared to *Smarcb1*^+/+^ mice and develop enlarged ventricles detectable in MRI with a penetrance of 100%. Among them, 60% of mutant animals develop a severe phenotype with visible hydrocephalus within 50 days after birth. Enlarged ventricles and thinner cerebral cortices can only be observed in adolescent *Smarcb1*^1148del/1148del^ mice. The excess of CSF can neither be explained by hyperproliferation of the choroid plexus nor by tumour growth. Thus, we concluded that the development of enlarged lateral ventricles takes place within the first 4 weeks of life. Therefore, we decided to further investigate newborn mice for indications of anomalies in brain development at the transcriptomic level.

### Single-Cell Transcriptomics Shows the Cellular Heterogeneity of *Smarcb1*^+/+^ and *Smarcb1*^1148del/1148del^ Mouse Brains

**Fig. 3:**
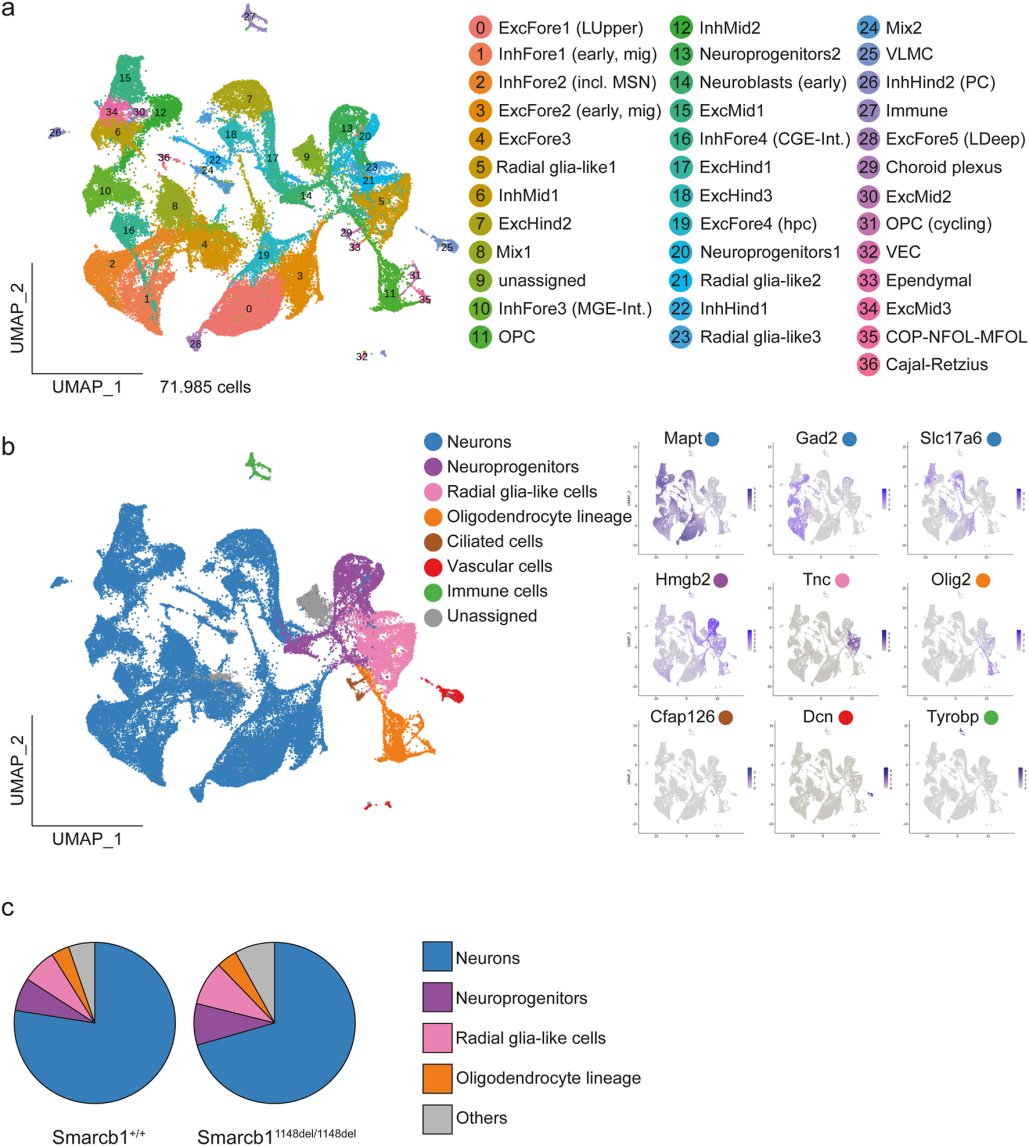
Single-cell transcriptomes in wild-type and *Smarcb1*^1148del/1148del^ mouse brains. **a** UMAP plot showing the distribution of distinct cell types after integration of single-cell transcriptomes from brain cells of two wild-type and two *Smarcb1*^1148del/1148del^ P0 mice. **b** Assignment of seven distinct cell classes; for each class, the expression of one typical marker gene is shown on the right. **c** Pie chart showing the distribution of cell classes in the *Smarcb1*^+/+^ brain (left) and in the *Smarcb1*^1148del/1148del^ brain (right). *Fore* forebrain, *Mid* midbrain, *Hind* hindbrain, *Exc* excitatory neurons, *Inh* inhibitory neurons, M*ix* neurons with mixed neurotransmitters, *Int* interneurons, *OPC* oligodendrocyte precursor cells, *COP* committed oligodendrocyte precursors, *NFOL* newly formed oligodendrocytes, *MFOL* myelin-forming oligodendrocytes, *VLMC* vascular and meningeal cells, *VEC* vascular endothelial cells, *LUpper* upper layers, *LDeep* deep layers, *MSN* medium spiny neurons, *mig* migrating, *PC* purkinje cells, *hpc* hippocampal, *CGE* caudal ganglionic eminence, *MGE* medial ganglionic eminence, *n*.*s*. not significant, *P0* postnatal day 0 (day of birth)

We performed scRNA-seq of cells isolated from the brains of two wild-type and two *Smarcb1*^1148del/1148del^ animals at their day of birth (P0) using the 10X Genomics platform. Brains were cut between the mid- and hindbrain into supra- and infratentorial parts, which were further processed and sequenced separately (Online Resources 7a, 8). After pre-processing and quality control, the eight samples with a total of 71,985 cells were integrated and projected onto a two-dimensional map, on which wt and mt cells were evenly distributed (Fig. 3a, Online Resource 7b). We identified 37 clusters representing distinct cell types (Fig. 3a, Online Resource 9). Differentially expressed gene analyses and a cell cycle state plot enabled us to annotate each cluster by the dominant cell type (Fig. 3a, Online Resources 7c, 9, 10, 11). As a reference, we used a comprehensive transcriptomic single-cell atlas of the Developing Mouse Brain (La Manno et al. 2021; www.mousebrain.org/development) and complementary publications for embryonic and perinatal brain development (Carter et al. 2018; Loo et al. 2019). Each cluster was assigned to one of seven superordinate cell classes (Fig. 3b), except for cluster 9, where a low number of unique molecular identifiers and a low gene count prevented this. Cells of all clusters express *Smarcb1* (Online Resource 7d).

The majority of cells are post-mitotic neurons, which can be subdivided into excitatory (*Slc17a6*) and inhibitory (GABAergic: *Gad2*; glycinergic: *Slc6a5)* neurons (Fig. 3b, Online Resource 10). Forebrain excitatory neurons include migrating neurons (cl. 3: *Sema3c*, *Unc5d*), neurons of the upper (cl. 0: *Satb2*, *Pou3f2*) and deeper cortex layers (cl. 28: *Tle4*, *Hs3st4*) and developing hippocampal neurons (cl. 19: *Crym*, *Nrp2*) (Wiegreffe et al. 2015; Loo et al. 2019; La Manno et al. 2021). Mid- and hindbrain excitatory neurons are distributed in clusters 15, 30, and 34 (*Tcf7l2*, *Ebf2*) and 7, 17, and 18 (*Nhlh1*, *Neurod1*) (Zeisel et al. 2018; Carter et al. 2018; La Manno et al. 2021). Inhibitory forebrain neurons (*Dlx1*) include migrating neurons (cl. 1: *Tiam2*, *Sp8*), medium spiny neurons (cl. 2: *Foxp1*, *Bcl11b)*, medial ganglionic eminence-derived interneurons (cl. 10: *Lhx6*, *Ackr3*) and caudal ganglionic eminence-derived interneurons (cl. 16: *Adarb2*, *Htr3a*) (Wei et al. 2019; Lindtner et al. 2019; Loo et al. 2019; La Manno et al. 2021). In addition, midbrain inhibitory neurons (cl. 12: *Sox14*, *Asic4*), caudal hindbrain neurons (cl. 22: *Tfap2a*, *Skor1*) and Purkinje cells (cl. 28: *Car8*, *Pcp2*) can be distinguished (Carter et al. 2018; La Manno et al. 2021). Finally, two clusters (8, 24) contain a mix of both excitatory and inhibitory cells.

Neuroprogenitors include cycling neuroprogenitors (cl. 20, 13: *Mki67*, *Hmgb2*) and neuroblasts (cl.14: *Mafp4*, *Ebf3*) (Online Resources 7c, 11a) (Tirosh et al. 2016; La Manno et al. 2021). Radial glia-like cells (cl. 5, 21, 23: *Tnc*, *Pla2g7*) give rise to oligodendrocytes (OL), astrocytes and ependymal cells (Online Resource 11a) (La Manno et al. 2021). OL development (*Olig2)* is reflected by two clusters containing cycling (cl. 31) and non-cycling (cl. 11) oligodendrocytes precursor cells (*Pdgfra*, *Cspg4)*, and one cluster (cl. 25) that harbours more differentiated OL types such as committed oligodendrocyte precursors (*Gpr17*, *Bmp4*), newly-formed oligodendrocytes (*Tcf7l2)* and myelin-forming oligodendrocytes (*Plp1*, *Mbp)* (Online Resource 11a) (Marques et al. 2016; La Manno et al. 2021).

Ciliated cells (*Cfap126*) comprise cells of the choroid plexus (cl. 29: *Ttr*, *Kcne2*) and ependymal cells (cl. 33: *Ccdc153*, *Meig1*) (Online Resource 11b) (Gegg et al. 2014; La Manno et al. 2021). The vascular clusters include vascular and leptomeningeal cells (cl. 25: *Dcn*, *Lum*) and endothelial cells (cl. 32: *Cldn5*, *Flt1)* (Online Resource 11b) (La Manno et al. 2021). Immune cells (cl. 27: *Tyrobp*) comprise macrophages (*Pf4*, *Mrc1*), microglia (*Aif1*, *Hexb*), and mononuclear cells (*C1qb*, *Fcer1g*) (Online Resource 11b) (La Manno et al. 2021).

A comparison of the proportion of wild-type and mutant cells in the different cell classes revealed a slight shift from neurons to neuroprogenitors in the mutant brain (Fig. 3c). However, no significant difference between wild-type and mutant samples was found (Online Resource 7e).

These analyses demonstrate that at the time of birth the brains of *Smarcb1*^1148del/1148del^ mice still reflect the entirety of all cell types in a physiologic mouse brain. From this, we deduce that despite the SMARCB1 mutation, a complete brain including all cell types can be formed.

### Single-Cell transcriptomes of *Smarcb1*^1148del/1148^^del^ neurons revealed altered signalling pathways

**Fig. 4:**
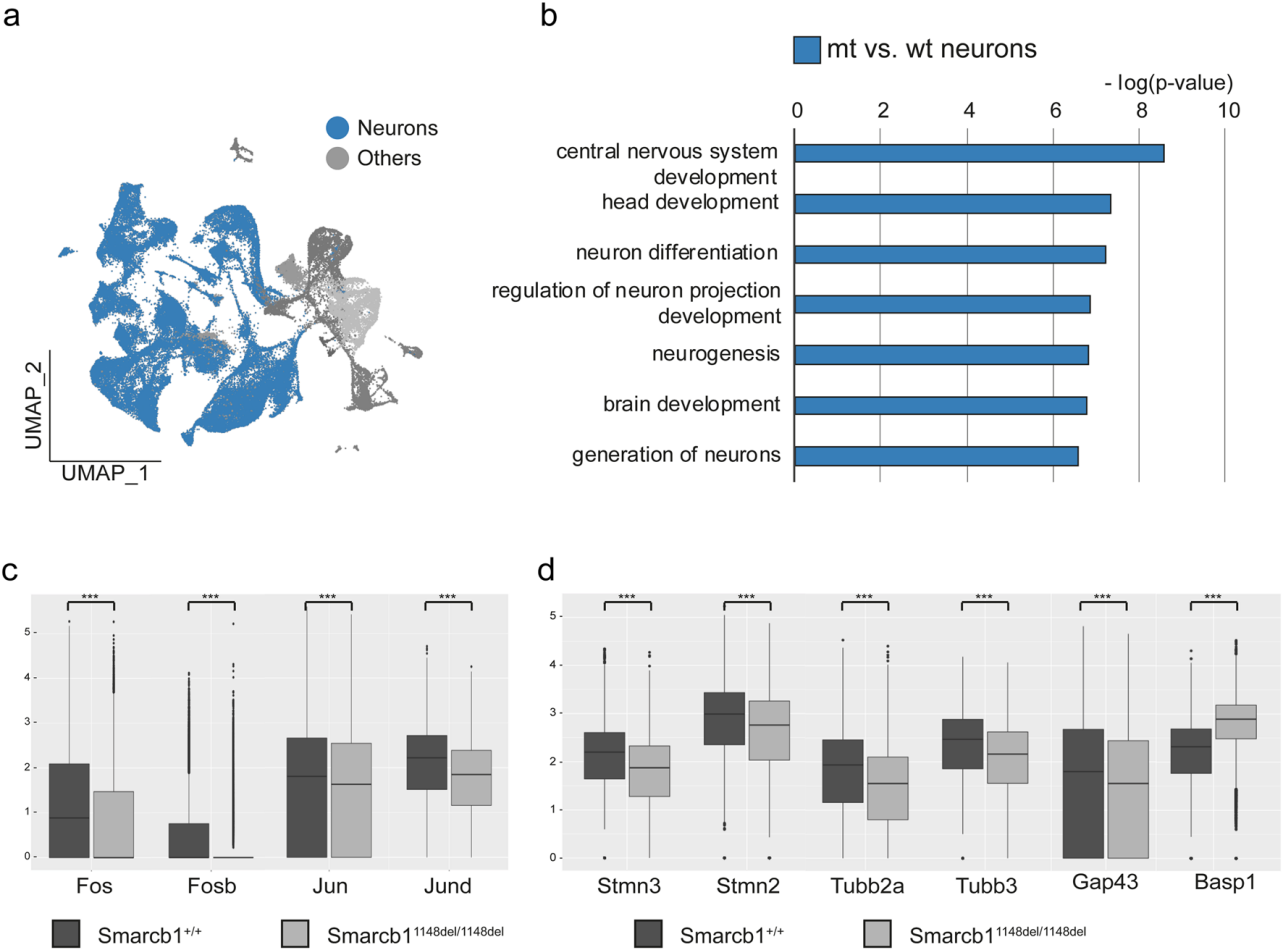
Comparison of the transcriptome of *Smarcb1*^+/+^ and *Smarcb1*^1148del/1148del^ brain cells. **a** Merged UMAP of brain cells. All neurons are indicated in blue. **b** Bar chart showing the enrichment of biological processes related to neurogenesis and neuron development in *Smarcb1*-mutant neurons compared to neurons in wild-type animals, as revealed by gene ontology analysis using ToppGene Suite (Chen et al. 2009; https://toppgene.cchmc.org/) **c** expression of AP-1 transcription factor family members, and **d** of neurite outgrowth-associated genes, in *Smarcb1*-mutant and wild-type neurons. *** Indicates *p* ≤ 0.001 (two-sided unpaired *t*-test, Bonferroni correction applied)

Next, we focussed on the expression pattern of neuronal clusters forming the largest cell class at time point P0 (Fig. 4a). We compared neurons of *Smarcb1* wt and mt brains by performing gene ontology analysis (Chen et al. 2009). In *Smarcb1* mutant neurons, we detected an enrichment of transcription factors (e.g. *Neurod2*, *Lhx2*, *Sox11*) and biological processes connected to neuronal development (Fig. 4b). Moreover, several genes encoding for activator protein 1 (AP-1) transcription factor family members, such as *Fos*, *Fosb*, *Jun*, and *Jund*, showed significantly reduced expression levels in mutant neurons (Fig. 4c).

Furthermore, *Smarcb1*^1148del/1148del^ neurons showed a significantly lower expression of several important factors involved in neurite outgrowth, such as Stathmins (*Stmn2* and *Stmn3*), Tubulins (*Tubb2a* and *Tubb3*), or *Gap43* (Fig. 4d). While stathmins and tubulins are needed for microtubule organization in axons, dendrites and synapses, GAP43 regulates actin dynamics in neuronal growth cones (Hartl and Schneider 2019). Interestingly, *Basp1*, which can partly replace the function of *Gap43* in neurite outgrowth (Korshunova et al. 2008), is upregulated in *Smarcb1*^1148del/1148del^ neurons (Fig. 4d). This points to an altered formation of neurites and axons in mutant neurons, which has already been described for other C-terminal mutations of *SMARCB1* (Valencia et al. 2019) as well as for mutations of other BAF subunits, i.e. for *Arid1b* and *Actl6b* (Wu et al. 2007; Ka et al. 2016; Jung et al. 2017).

## Discussion

From the broad spectrum of *SMARCB1* mutations, we investigated the C-terminal *Smarcb1* c.1148del mutation in a newly established mouse model. Homozygous mutant mice showed delayed weight gain and enlarged lateral ventricles in MRI and histology. This phenotype was not found in heterozygous mice. ScRNA-seq revealed the differentiation of progenitor cells into all expected cell types but altered AP-1 and neurite outgrowth signalling pathways in *Smarcb1*^1148del/1148del^ neurons.

The human *SMARCB1* c.1148 cytosine point deletion (*SMARCB1* c.1148del/p.P383RfsX100 (COSM1057)) is closely associated with the formation of ATRT of the central nervous system and was also reported in tumours of the soft tissue and the thyroid gland (Tate et al. 2019). ATRT with this mutation are SMARCB1-negative on IHC staining, indicating that the frameshift of 100 amino acids in the mutated human protein causes unstable or misfolded proteins (Tsai et al. 2012). In contrast, no tumour formation was detected in *Smarcb1*^1148del/1148del^ mice and in IHC stainings, all cells were SMARCB1-positive. The cause for this deviant phenotype might be that the same mutation at DNA level leads to a frameshift of only 36 amino acids in mutated murine proteins, resulting in an elongated SMARCB1 protein that might still be partially functional.

Mutations of the *Smarcb1* C-terminal region can predispose to the neurodevelopmental disorder Coffin–Siris syndrome (Holsten et al. 2018;). Valencia et al. (2019) hypothesize that this is due to disruption of the binding between the coiled-coil C-terminal domain and the nucleosome acidic patch. Most Coffin–Siris syndrome characteristics such as microcephaly and abnormal corpus callosum did not apply to *Smarcb1*^1148del/1148del^ mice. Shared abnormalities were growth delay, which has been observed especially in *SMARCB1* pathogenic variants (Santen et al. 2013), and hydrocephalus, which has only recently been described as a common prenatal characteristic in *ARID1A* pathogenic variants (but not in *SMARCB1* variants) (van der Sluijs et al. 2022).

The most striking characteristic of *Smarcb1*^1148del/1148del^ mice is hydrocephalus development. In general, hydrocephalus can be classified into acquired hydrocephalus due to haemorrhage, infection or neoplasm, and congenital/developmental hydrocephalus (CH) without any extrinsic cause (Tully and Dobyns 2014). Studies of CH have led to the identification of several pathogenic human genes (Tully and Dobyns 2014; McKnight et al. 2021), including genes of the BAF complex: *de novo* and transmitted *SMARCC1* mutations were identified in CH patients with stenosis of the aqueduct (Furey et al. 2018; Jin et al. 2020;). Diets et al. (2019) described a *SMARCB1* mutation in exon 2 (c.110G > A; p.Arg37His) that causes intellectual disability and hydrocephalus due to choroid plexus hyperplasia. In addition, BAF mouse models showing hydrocephalus were established for *Smarca4/Brg1* (Cao and Wu 2015; Holdhof et al. 2020) and *Arid1b* (Celen et al. 2017; Shibutani et al. 2017).

In our mouse model, *Smarcb1*^1148del/1148del^ mice developed enlarged ventricles visible in MRI in all cases and externally visible hydrocephalus with a penetrance of 60%. Histology revealed an unremarkable choroid plexus and no evidence of tumour formation, inflammation, or haemorrhage. Since P0 brains show no reduced neuron count in neither histology nor scRNA-seq, we conclude that *hydrocephalus e vacuo* is unlikely. MRI indicates that obstruction of the aqueduct might be causing the hydrocephalus. We interpret this as an indication that *Smarcb1*^1148del/1148del^ mice might develop congenital non-communicating hydrocephalus within their first 50 days of life, whereby the incomplete penetrance might be a consequence of varying degrees of restricted CSF circulation. Further investigation is needed to understand the exact aetiology.

Apart from hydrocephalus and thinned cortices in adolescent mice, we could not observe any further structural brain abnormalities in *Smarcb1*^1148del/1148del^ mice. Therefore, the *Smarcb1* c.1148del mutation seems to have a rather small influence on murine brain development compared to other *Smarcb1* mutations, which have been described to cause a broad variety of brain abnormalities. For example, the biallelic loss of *Smarcb1* in defined cell populations was described to cause embryonic lethality, rhabdoid tumours or cerebellar hypoplasia (Moreno et al. 2014; Graf et al. 2022). Heterozygous *Smarcb1* disruption in neural stem/progenitor cells led to microcephaly, brain midline abnormalities and choroid plexus hyperplasia (Filatova et al. 2019). None of these severe neurodevelopmental malformations were present in *Smarcb1*^1148del/1148del^ mice, suggesting that the function of SMARCB1 within the BAF complex is largely preserved.

Although the *Smarcb1* c.1148del mutation still allows the formation of a complete brain, we hypothesized that it might alter protein interactions and thereby affect cell differentiation and/or signalling pathways in developing neural cells. To investigate these differences triggered by the mutation on a transcriptomic level, we performed scRNA-seq on two wild-type and two mutant P0 mouse brains.

The comparison of wild-type and mutant brains revealed a similar distribution and similar numbers for all cell classes. Only a slight non-significant shift from neurons to neuronal progenitor cells could be observed. Hence, a similar block of neuronal differentiation as observed in cerebral organoids upon knockdown of *SMARCB1* during early neural differentiation (Parisian et al. 2020) could not be detected. However, neuron differentiation in mutant brains might be somewhat delayed, since we observed an enrichment of biological processes and transcription factors connected to neuronal development in the mutant post-mitotic neurons.

Moreover, our dataset revealed two important signalling pathways affected by the mutation. First, we showed a lower expression of AP-1 transcription factors in mutant neurons. These factors include various protein dimers made up of the members of the Jun, Fos, Maf and ATF families (Bejjani et al. 2019). FOS/JUN dimers were shown to interact with the SMARCD1 subunit of the BAF complex (Ito et al. 2001), and probably recruit the BAF complex in order to mediate chromatin accessibility (Vierbuchen et al. 2017). This close relationship seems to be influenced by the *Smarcb1* c.1148del mutation, resulting in lower levels of AP-1 transcription factors, which in turn might alter related neuronal functions, including neuronal differentiation (Pagin et al. 2021), neurite outgrowth (Dragunow et al. 2000; Jessen et al. 2001; Seijffers et al. 2006), and response to neuronal-activity (Su et al. 2017).

Second, we detected a reduced expression of factors that are important for neurite outgrowth via growth cones. These include Stathmin and Tubulin, which participate in microtubule organization, and growth-associated protein-43 (Gap43) and brain acid-soluble protein 1 (Basp1), which are involved in neurodevelopment, synaptic function and nerve regeneration (Chung et al. 2020). In *Smarcb1* mutant neurons, decreased levels of *Stmn3*, *Stmn2*, *Tubb2a* and *Tubb3* suggest a reduced promotion of neurite outgrowth. *Basp1* levels, on the other hand, were increased, which suggests an alternative activation route to compensate for low *Gap43* levels. It is known that BASP1 can substitute GAP43 in terms of the induction of NCAM-independent neurite outgrowth, but not NCAM-mediated neurite outgrowth (Korshunova et al. 2008). The altered expression levels could possibly be a consequence of reduced AP-1 signalling, as ATF-3 (Seijffers et al. 2006), c-Jun (Dragunow et al. 2000) and c-Fos (Jessen et al. 2001) have been shown to promote neurite outgrowth.

Our findings are in line with previous studies showing neurite outgrowth deficits in neurons with BAF mutations. Differentiated neurons derived from induced pluripotent stem cells harbouring a heterozygous *SMARB1* p.K364del mutation showed less neurite outgrowth than wild-type controls (Valencia et al. 2019). *Actl6b*^−/−^ hippocampal neurons displayed shorter and less complex dendritic growth (Wu et al. 2007). *Arid1b* haploinsufficiency in cortical and hippocampal pyramidal neurons caused fewer and shorter dendritic spines. Additionally, decreased levels of neurite-associated transcripts (*Stmn2*, *Gap43*), *Arc* and the AP-1 transcription factor *c-Fos* were observed, just as in our mouse model. Ka et al. (2016), therefore, hypothesize that ARID1B participates in neurotrophin-mediated *c-Fos* and/or *Arc* expression and thereby regulates dendritic development. These findings suggest that a well-functioning BAF complex including a functional DNA-binding SMARCB1 protein is crucial for neurite outgrowth and dendritic development and allows for an appropriate reaction to external stimuli. Impaired SMARCB1 function could disturb neurite outgrowth and synapse formation and may lead to intellectual disability.

Overall, in this study, we have presented the establishment of a new mouse model with a *Smarcb1* c.1148del mutation. We have shown that this mutation leads to hydrocephalus and weight loss in homozygous mice. Using scRNA-seq, we have illustrated that although the mutation still allows the formation of differentiated cell types, it interferes with important signalling pathways such as AP-1 transcription factors and neurite outgrowth. This study thus confirms the crucial role of SMARCB1 in neuronal development and in the pathogenesis of neurodevelopmental disorders.

## Supplementary Information

Below is the link to the electronic supplementary material.

Supplementary material 1 (PDF 3862.9 kb)

Supplementary material 2 (XLSX 55.2 kb)

## Data Availability

The single-cell RNA sequencing data generated in this study have been deposited in NCBI’s Gene Expression Omnibus (Edgar et al. 2002) and are accessible through GEO Series accession number GSE212672, https://www.ncbi.nlm.nih.gov/geo/query/acc.cgi?acc=GSE212672.
