# Supplementary material for: A Carboxy-terminal Smarcb1 Point Mutation Induces Hydrocephalus Formation and Affects AP-1 and Neuronal Signalling Pathways in Mice
Source: Cell Mol Neurobiol. 2023 May 23;43(7):3511–26. doi: 10.1007/s10571-023-01361-5 (PMC10477118; doi:10.1007/s10571-023-01361-5)
Supplement: Supplementary file 1 — Supplementary material 1 (PDF 3862.9 kb) [file 10571_2023_1361_MOESM1_ESM.pdf]

A carboxyterminal *Smarcb1* point mutation induces hydrocephalus formation and affects AP-1 and neuronal signalling pathways in mice

Cellular and Molecular Neurobiology

Aliska K. Brugmans[1,*], Carolin Walter[1,2,*], Natalia Moreno[1], Carolin Göbel[3,4,5], Dörthe Holdhof[3,4,5], Flavia W. de Faria[1], Marc Hotfilder[1], Daniela Jeising[1], Michael C. Frühwald[6] (ORCID 0000-0002-8237-1854), Boris V. Skryabin[7], Timofey S. Rozhdestvensky[7], Lydia Wachsmuth[8], Cornelius Faber[8], Martin Dugas[2,9], Julian Varghese[2], Ulrich Schüller[3,4,5], Thomas K. Albert[1,§], Kornelius Kerl[1,§]

[1] Department of Paediatric Haematology and Oncology, University Children's Hospital Münster, 48149 Münster, Germany

[2] Institute of Medical Informatics, University of Münster, 48149 Münster, Germany

[3] Department of Paediatric Haematology and Oncology, University Medical Center Hamburg-Eppendorf, 20251 Hamburg, Germany

[4] Research Institute Children's Cancer Center, 20251 Hamburg, Germany

[5] Institute of Neuropathology, University Medical Center Hamburg-Eppendorf, 20251 Hamburg, Germany

[6] Swabian Children's Cancer Center, Paediatrics and Adolescent Medicine, University Medical Center Augsburg, 86156 Augsburg, Germany

[7] Medical Faculty, Core Facility TRAnsgenic Animal and Genetic Engineering Models (TRAM), University of Münster, 48149 Münster, Germany

[8] Clinic of Radiology, Translational Research Imaging Center (TRIC), University of Münster, 48149 Münster, Germany

[9] Institute of Medical Informatics, Heidelberg University Hospital, 69120 Heidelberg, Germany

[*] **These authors contributed equally to this work.**

[§] **These authors are shared senior authors.**

Corresponding author: Kornelius Kerl; kornelius.kerl@ukmuenster.de

Online Resource 1

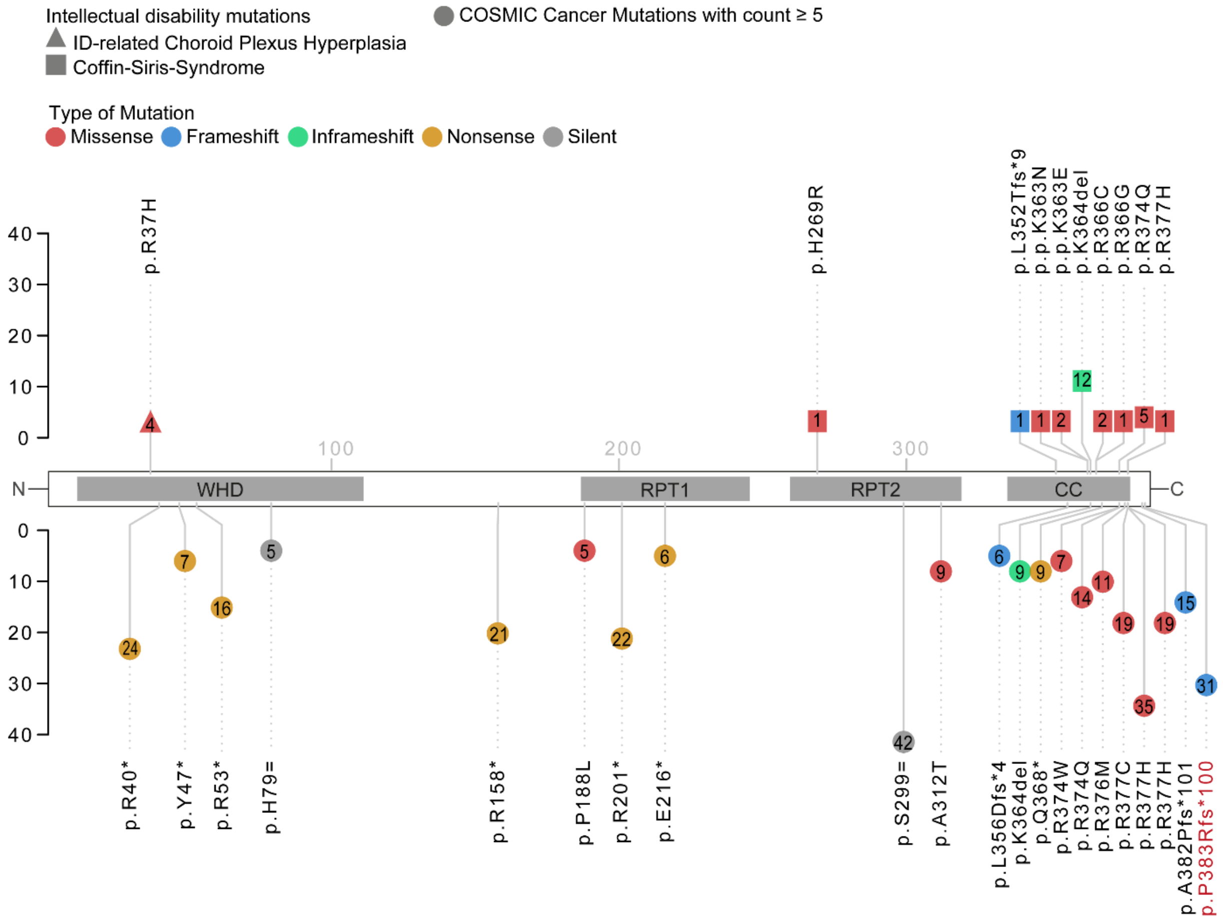

Online Resource 1 (related to Figure 1): Mutational Spectrum of *SMARCB1*

Scheme illustrating the mutational spectrum of human *SMARCB1* (ENST00000644036). Upper half shows the mutations of intellectual disability (ID) syndromes: ID-related choroid plexus hyperplasia (triangle) (Kleefstra et al. 2012; Diets et al. 2019) and Coffin-Siris-Syndrom (square) (Tsurusaki et al. 2012, 2014; Santen et al. 2013; Wieczorek et al. 2013; Gossai et al. 2015; Filatova et al. 2019; Sekiguchi et al. 2019; Cheng et al. 2021). Bottom half shows frequent cancer mutations (circle) (count ≥ 5, COSMIC, cancer.sanger.ac.uk) (Tate et al. 2019). Colours indicate type of mutation (red, missense; blue, frameshift; green, frameshift; yellow, nonsense; grey, silent). Y-Axis position and number in the node indicate count. Human *SMARCB1* c.1148del/p.P383RfsX100 (COSM1057) is indicated in red. WHD, winged helix DNA binding; RPT1, repeat 1; RPT2, repeat 2; CC, coiled-coil domain; CNS, central nervous system

Online Resource 2

Referral Diagnosis	AA Position	CDS Mutation	AA Mutation	Count	Shift	Reference
CSS	269	c.806A>G	p.H269R	1	Missense	(Sekiguchi et al. 2019)
CSS	352	c.1052dup	p.L352Tfs*9	1	Frameshift	(Sekiguchi et al. 2019)
CSS	363	c.1089G>T	p.K363N	1	Missense	(Santen et al. 2013)
CSS	363	c.1087A>G	p.K363E	2	Missense	(Sekiguchi et al. 2019) (Lee et al. 2021)
CSS	364	c.1091_1093del	p.K364del	12	Inframeshift	(Tsurusaki et al. 2012) (Santen et al. 2013) (Tsurusaki et al. 2014) (Filatova et al. 2019) (Sekiguchi et al. 2019)
CSS	366	c.1096C>T	p.R366C	2	Missense	(Wieczorek et al. 2013) (Cheng et al. 2021)
CSS	366	c.1096C>G	p.R366G	1	Missense	(Sekiguchi et al. 2019)
CSS	374	c.1121G>A	p.R374Q	5	Missense	(Wieczorek et al. 2013) (Gossai et al. 2015) (Filatova et al. 2019) (Sekiguchi et al. 2019)
CSS	377	c.1130G>A	p.R377H	1	Missense	(Tsurusaki et al. 2012)
ID-CPH	37	c.110G>A	p.R37H	4	Missense	(Kleefstra et al. 2012) (Diets et al. 2019)
Cancer	40	c.118C>T	p.R40*	24	Nonsense	Cosmic Cancer (last access 08/08/2022), COSM1002
Cancer	47	c.141C>A	p.Y47*	7	Nonsense	Cosmic Cancer (last access 08/08/2022), COSM991
Cancer	53	c.157C>T	p.R53*	16	Nonsense	Cosmic Cancer (last access 08/08/2022), COSM24595
Cancer	79	c.237C>T	p.H79=	5	Coding silent	Cosmic Cancer (last access 08/08/2022), COSM183186
Cancer	158	c.472C>T	p.R158*	21	Nonsense	Cosmic Cancer (last access 08/08/2022), COSM992
Cancer	188	c.563C>T	p.P188L	5	Missense	Cosmic Cancer (last access 08/08/2022), COSM110414
Cancer	201	c.601C>T	p.R201*	22	Nonsense	Cosmic Cancer (last access 08/08/2022), COSM993

Cancer	216	c.646G>T	p.E216*	6	Nonsense	Cosmic Cancer (last access 08/08/2022), COSM1004
Cancer	299	c.897G>A	p.S299=	42	Coding silent	Cosmic Cancer (last access 08/08/2022), COSM1009
Cancer	312	c.934G>A	p.A312T	9	Missense	Cosmic Cancer (last access 08/08/2022), COSM1226776
Cancer	356	c.1066_1067del	p.L356Dfs*4	6	Frameshift	Cosmic Cancer (last access 08/08/2022), COSM5967280
Cancer	364	c.1091_1093del	p.K364del	9	Inframeshift	Cosmic Cancer (last access 08/08/2022), COSM1180929
Cancer	368	c.1102C>T	p.Q368*	9	Nonsense	Cosmic Cancer (last access 08/08/2022), COSM997
Cancer	374	c.1120C>T	p.R374W	7	Missense	Cosmic Cancer (last access 08/08/2022), COSM1226778
Cancer	374	c.1121G>A	p.R374Q	14	Missense	Cosmic Cancer (last access 08/08/2022), COSM998
Cancer	376	c.1127G>T	p.R376M	11	Missense	Cosmic Cancer (last access 08/08/2022), COSM7457386
Cancer	377	c.1129C>T	p.R377C	19	Missense	Cosmic Cancer (last access 08/08/2022), COSM3972885
Cancer	377	c.1130G>A	p.R377H	35	Missense	Cosmic Cancer (last access 08/08/2022), COSM989
Cancer	377	c.?	p.R377H	19	Missense	Cosmic Cancer (last access 08/08/2022), COSM4166186
Cancer	382	c.1144del	p.A382Rfs*100	15	Frameshift	Cosmic Cancer (last access 08/08/2022), COSM1060
Cancer	383	**c.1148del**	**p.P383Rfs*100**	31	Frameshift	Cosmic Cancer (last access 08/08/2022), COSM1057

Online Resource 2 (related to Online Resource 1): Mutational Spectrum of SMARCB1

List of all Mutations of SMARCB1 mutations which have been reported for Coffin-Siris-Syndrome (CSS), intellectual disability-related choroid plexus hyperplasia (ID-CPH) and at the COSMIC Cancer Mutations database (only listed if count ≥ 5) (Tate et al. 2019). See corresponding Excel Table for a full list of mutations.

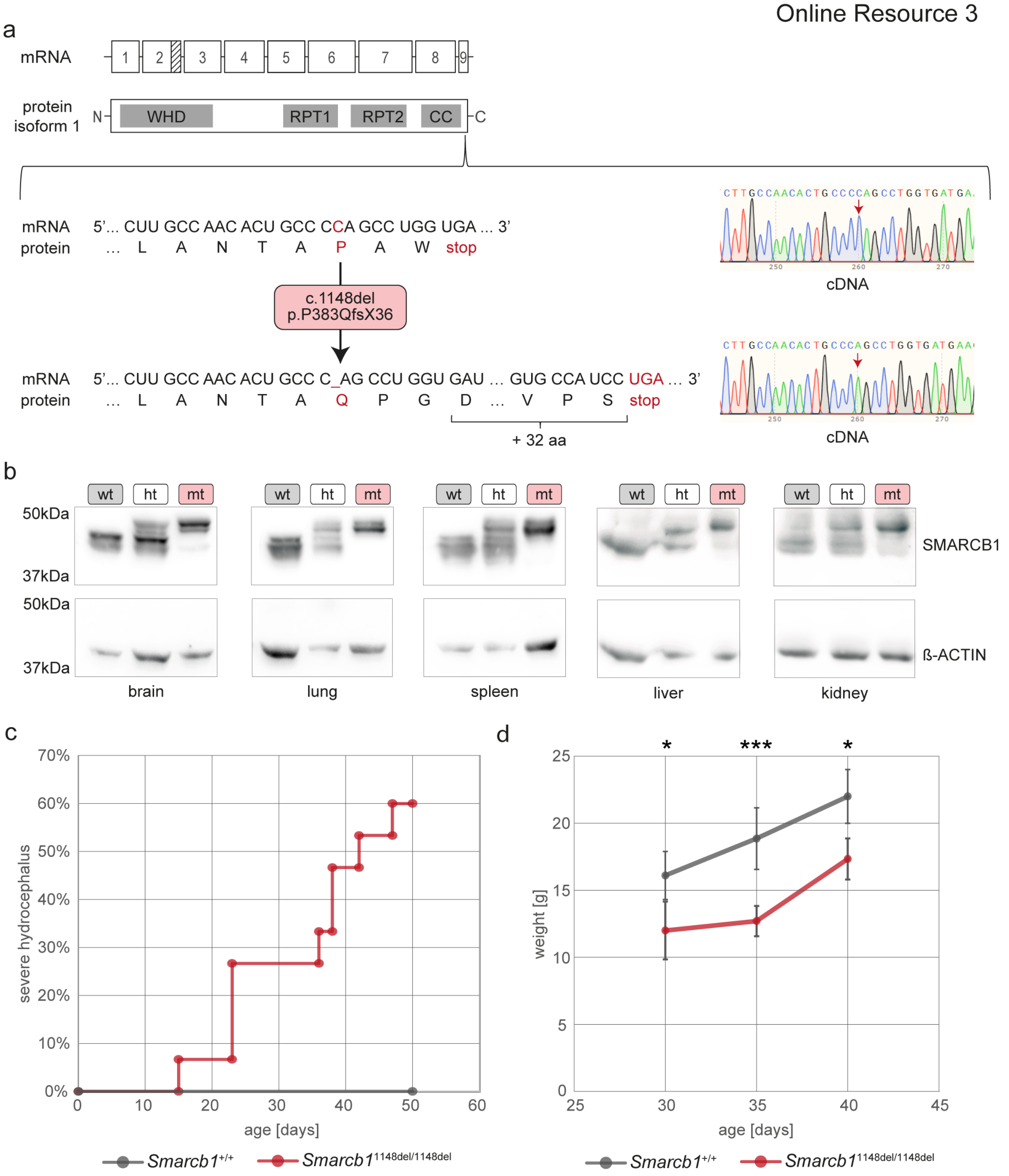

Online Resource 3 (related to Figure 1): Establishment of a *Smarcb1*$^{1148del/1148del}$ genetically engineered mouse model

(a) Schemes depicting the structure of murine *Smarcb1* wild-type mRNA (NM_011418.2) and the domain structure of isoform 1 of the SMARCB1 wt protein (top) and sequences of wild-type mRNA and protein sequence (middle). Sequences of *Smarcb1* c.1148del mRNA and

the resulting elongated SMARCB1 protein with 32 additional amino acids (bottom). Electropherograms on the right show the sequence of the wild-type allele (middle) and the mutant allele (bottom), with the deletion of cytosine 1148 indicated by an arrow.

(b) Immunoblot analysis of SMARCB1 protein expression in whole-cell lysates of the indicated organs in 12 weeks old mice of the genotype *Smarcb1*$^{+/+}$ (wt), *Smarcb1*$^{+/1148del}$ (ht), or *Smarcb1*$^{1148del/1148del}$ (mt).

(c) Detection rate of severe hydrocephalus development among *Smarcb1*$^{+/+}$ (n = 15) and *Smarcb1*$^{1148del/1148del}$ (n = 15) mice that were monitored for 50 days.

(d) Weight measurements of wild-type and *Smarcb1* mutant mice at postnatal day 30 (wt: 6 male, 4 female; mt: 2 male, 2 female), day 35 (wt: 6 male, 3 female; mt: 1 male, 2 female) and day 40 (wt: 3 male; mt: 2 male, 1 female). Measurements were subjected to a two-tailed unpaired t-test.

WHD, N-terminal winged helix DNA binding; RPT1, repeat 1; RPT2, repeat 2; CC = coiled-coil domain; aa, amino acids; del = deletion; fs = frameshift mutation; *, $p < 0.05$; ***, $p < 0.001$

Online Resource 4

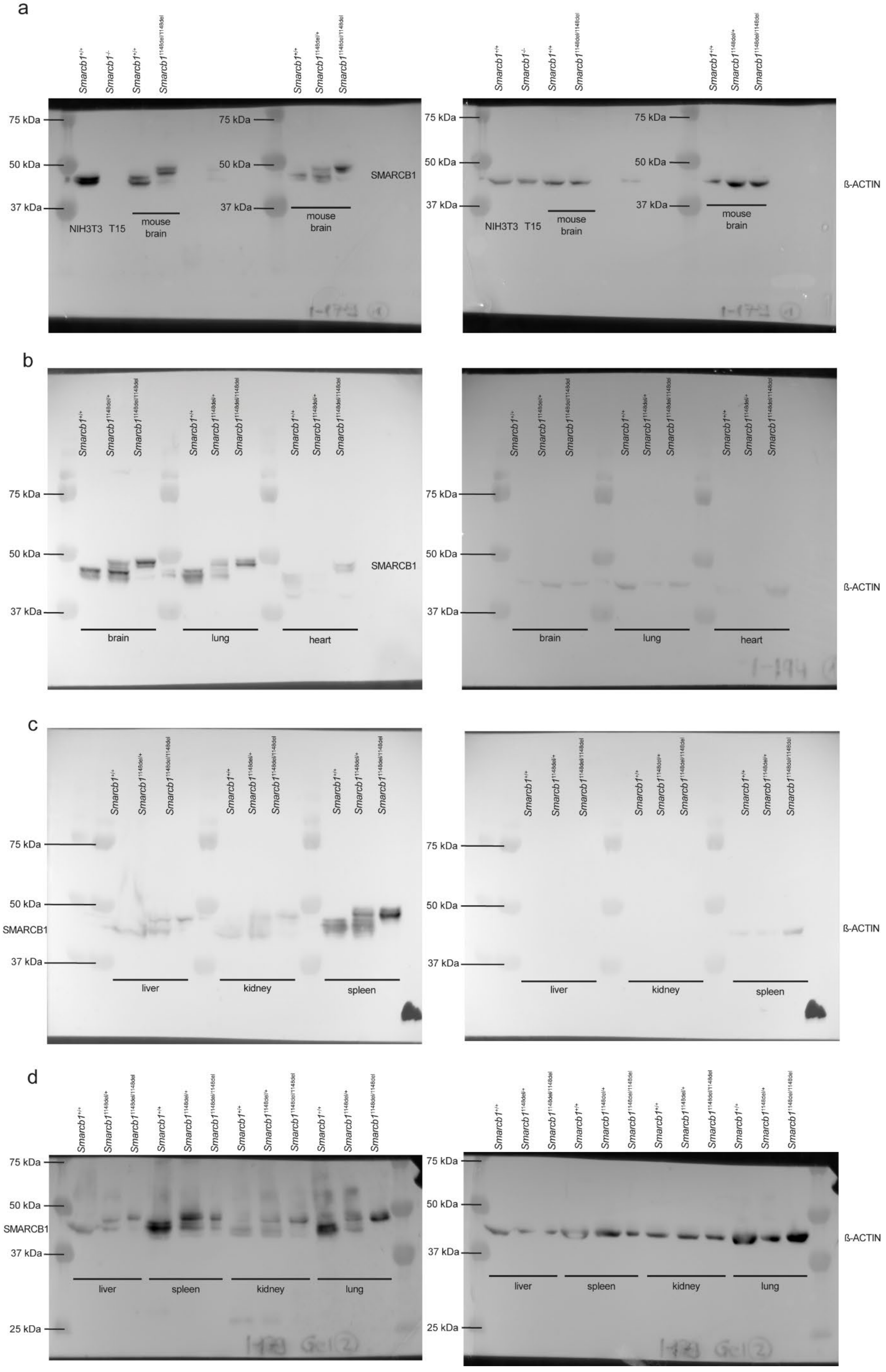

Online Resource 4 (related to Figure 1b and Online Resource 3b): Original Western Blots

(a) Full uncropped blots for **Figure 1b** using the SMARCB1 antibody (left: BD transduction laboratories, #612110, 1:1000) and the beta-actin antibody as a loading control on the same blot (right: Santa Cruz Biotechnology, #SC-47778, 1:10000).

(b) Full uncropped blots for **Online Resource 3b**, brain and lung, using the SMARCB1 antibody (left: Sigma Aldrich, #HPA018248, 1:200) and the beta-actin antibody as a loading control on the same blot (right: Santa Cruz Biotechnology, #SC-47778, 1:10000).

(c) Full uncropped blots for **Online Resource 3b**, spleen, using the SMARCB1 antibody (left: Sigma Aldrich, #HPA018248, 1:200) and the beta-actin antibody as a loading control on the same blot (right: Santa Cruz Biotechnology, #SC-47778, 1:10000).

(d) Full uncropped blots for **Online Resource 3b**, liver and kidney, using the SMARCB1 antibody (left: BD transduction laboratories, #612110, 1:1000) and the beta-actin antibody as a loading control on the same blot (right: Santa Cruz Biotechnology, #SC-47778, 1:10000).

Online Resource 5

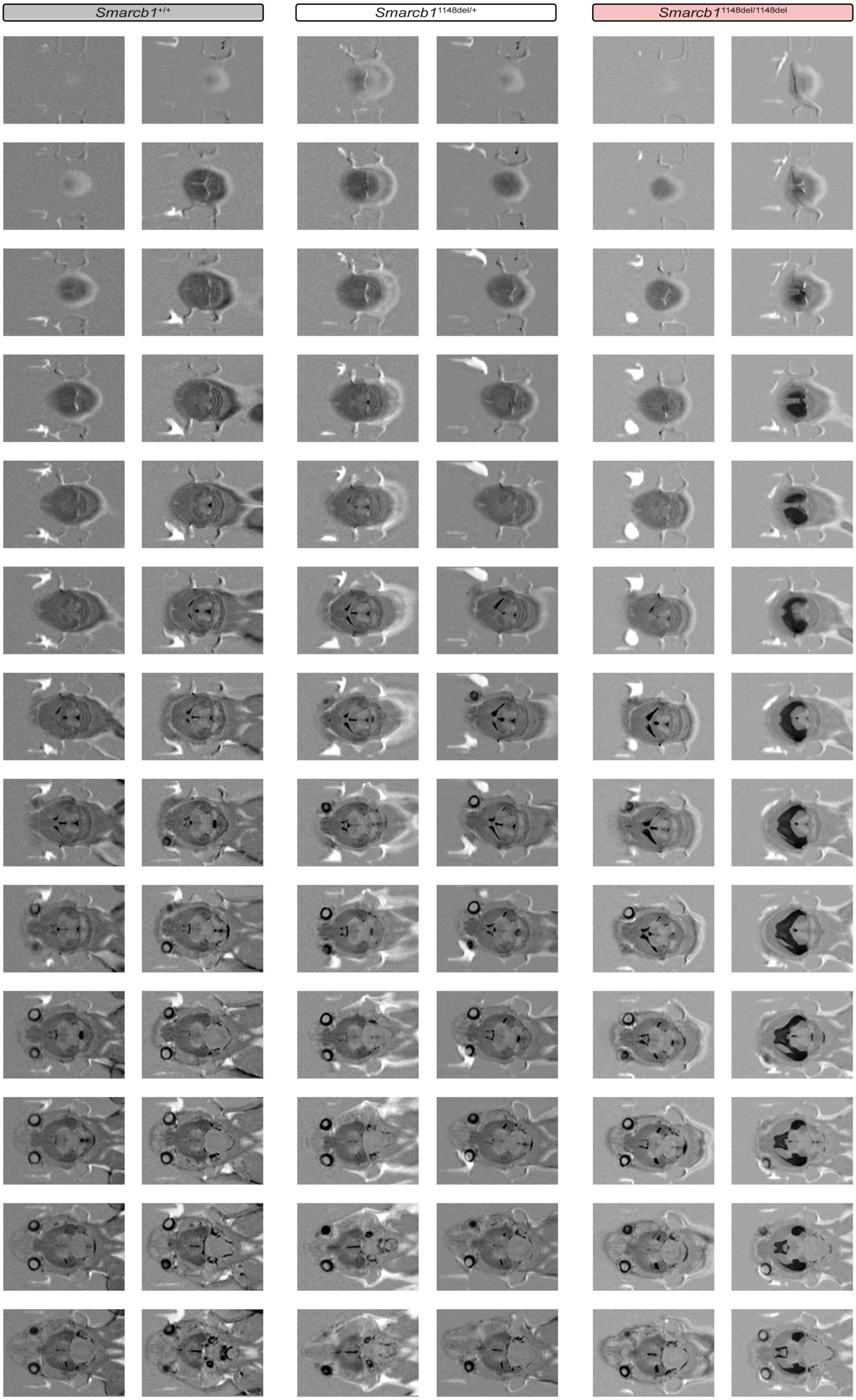

Online Resource 5 (related to Figure 1c): Magnetic resonance imaging

Exemplary magnetic resonance images of two *Smarcb1*$^{+/+}$, *Smarcb1*$^{1148del/+}$ and *Smarcb1*$^{1148del/1148del}$ mice each. Only the first 13 slices are shown.

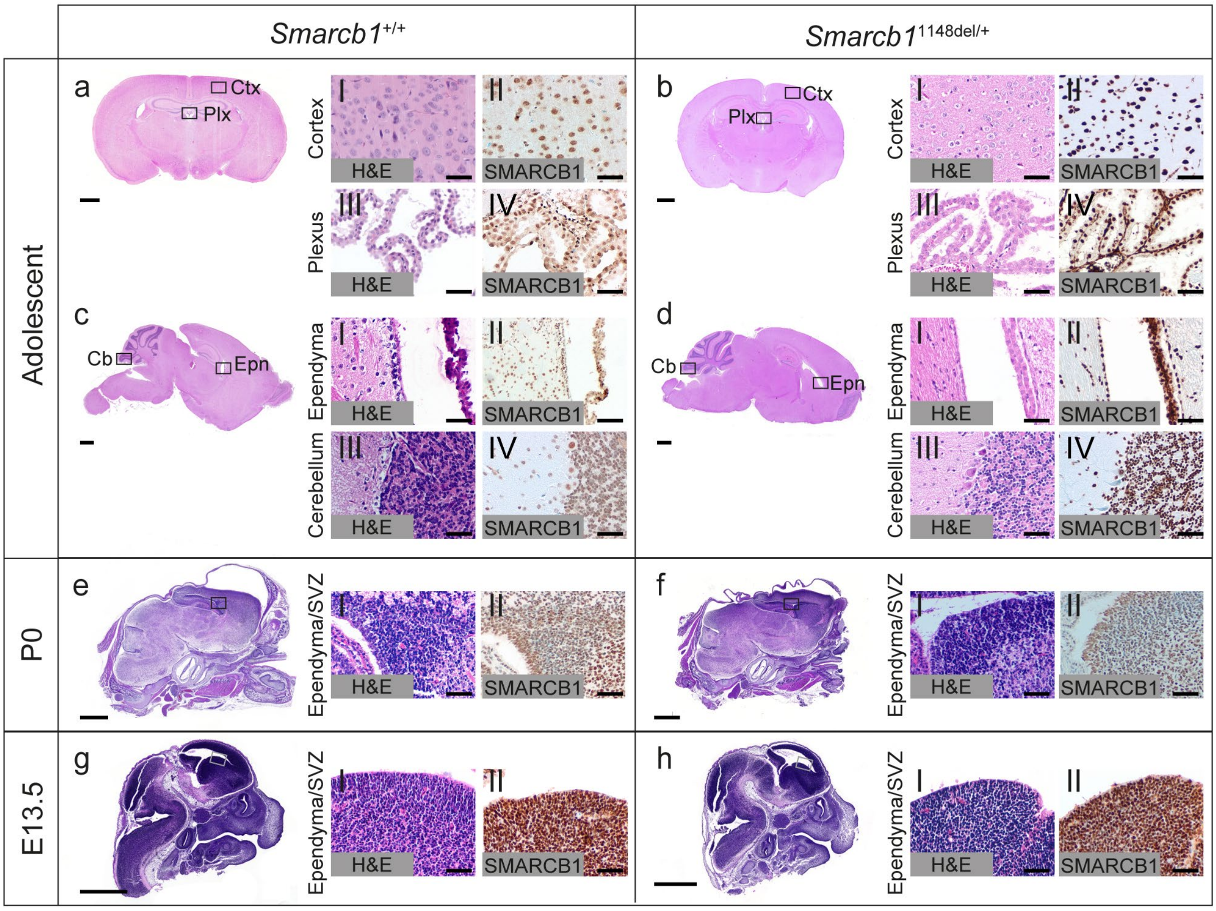

Online Resource 6 (related to Figure 2): Histological characterization of *Smarcb1*$^{1148del/+}$ mice

(a,b,c,d) Hematoxylin and Eosin (H&E) (I and III) and anti-SMARCB1 (II and IV) antibody stainings of brain sections from wild-type *Smarcb1*$^{+/+}$ (P30) (a, c) and heterozygous *Smarcb1*$^{1148del/+}$ (P84) (b, d) mutant mice. High-power images of the cortex (Ctx), the choroid plexus (Plx), the ependyma (Epn) and the cerebellum (Cb) are shown. n $\geq$ 3 animals.

(e, f) H&E (I) and anti-SMARCB1 (II) antibody stainings of brain sections from newborn (P0, day of birth) wild-type *Smarcb1*$^{+/+}$ (e) and heterozygous *Smarcb1*$^{1148del/+}$ (f) mice. High-power images of the ependyma including the subventricular zone (SVZ) are shown. n $\geq$ 3 animals.

(g, h) H&E (I) and anti-SMARCB1 (II) antibody stainings of brain sections from embryonal (E13.5) wild-type *Smarcb1*$^{+/+}$ (g) and heterozygous *Smarcb1*$^{1148del/+}$ (h) mice. High-power images of the ependyma including the SVZ are shown. n = 2 animals.

Scale bar: 1,000 µm in full brain images and 100 µm in high power images (20x magnification).

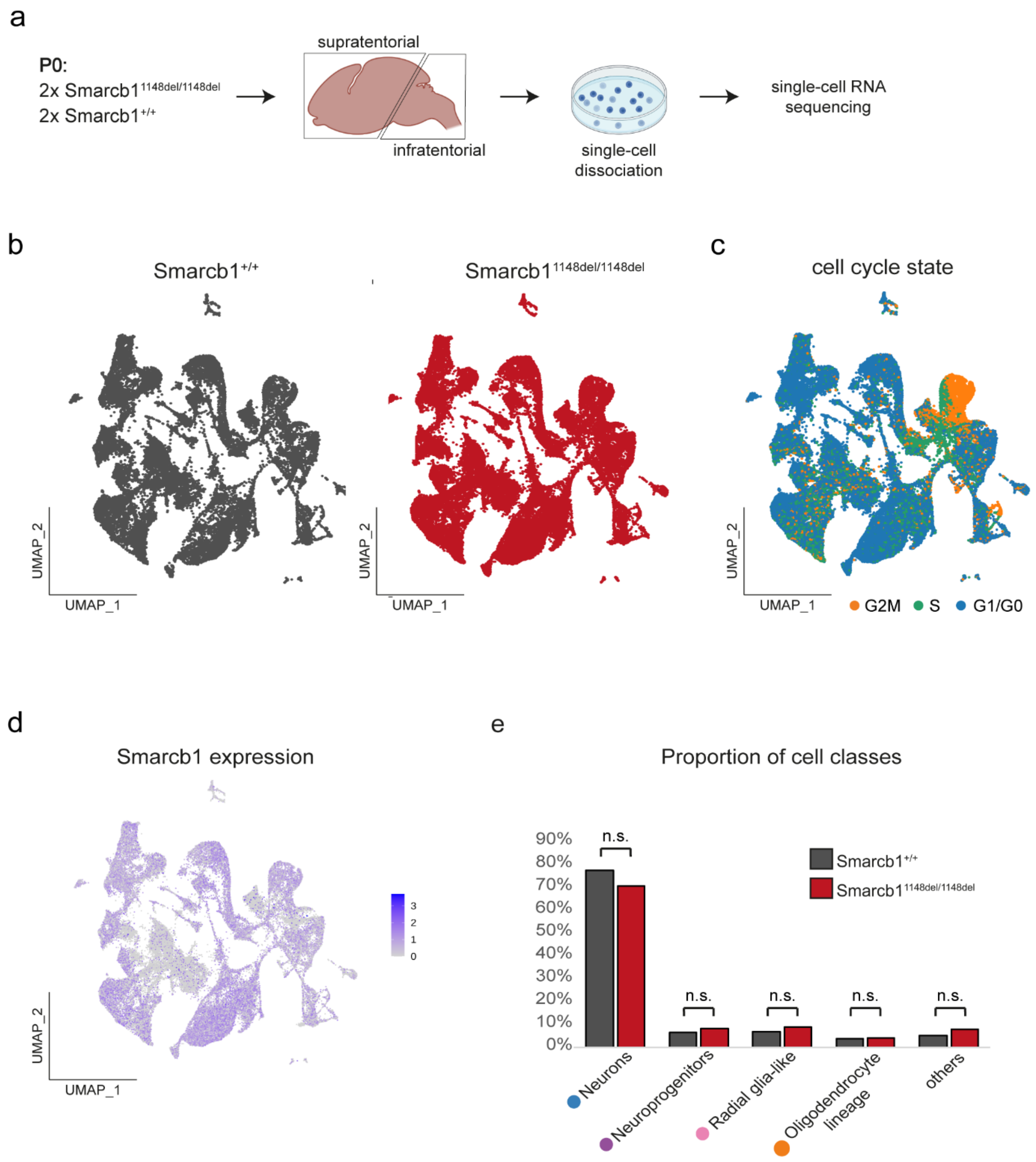

Online Resource 7 (related to Figure 3): scRNA-seq analysis of *Smarcb1*$^{+/+}$ and *Smarcb1*$^{1148del/1148del}$ mouse brains

(a) Flow scheme of the experimental set-up for single-cell transcriptomic analysis of wild-type and *Smarcb1*-mutant brain cells. Freshly prepared brains were initially separated into supratentorial and infratentorial parts, dissociated into single-cell suspensions, live cells

enriched by flow cytometry with 7-AAD staining, and finally subjected to scRNA-seq. Figure created with BioRender.com.

(b) UMAP maps showing even sample distribution of *Smarcb1*$^{+/+}$ cells (left) and *Smarcb1*$^{1148del/1148del}$ cells (right).

(c) UMAP map showing cell cycle states of individual cells

(d) UMAP map showing expression of murine *Smarcb1*, in individual cells.

(e) Bar chart showing the distribution of cell classes in wild-type and *Smarcb1*-mutant brains. Proportions were subjected to a Mann-Whitney U test.

P0, postnatal day 0 (day of birth); n.s., not significant

Online Resource 8

Mouse Model	Sample Name	Sex	Age [days]	Tissue	Estimated number of cells	Saturation [%]	Number of reads	Mean reads per cell	Median Genes per cell	Number of Cellls (after Quality Control)
$Smarcb1^{+/+}$	1i	Female	0	brain, infra-tentorial	4774	47.0	223614341	46840	2448	4560
$Smarcb1^{+/+}$	1s	Female	0	brain, supra-tentorial	7544	46.9	228168567	30245	1759	7309
$Smarcb1^{+/+}$	2i	Male	0	brain, infra-tentorial	7452	40.6	292365789	39233	3113	7038
$Smarcb1^{+/+}$	2s	Male	0	brain, supra-tentorial	8604	42.1	341664390	39709	2502	8323
$Smarcb1^{1148del/1148del}$	1i_Homo	Male	0	brain, infra-tentorial	10526	36.1	565542269	53728	3349	10112
$Smarcb1^{1148del/1148del}$	1s_Homo	Male	0	brain, supra-tentorial	12704	34.5	546843284	43044	2764	12465
$Smarcb1^{1148del/1148del}$	2i_Homo	Male	0	brain, infra-tentorial	9788	39.7	481875666	49231	3000	9602
$Smarcb1^{1148del/1148del}$	2s_Homo	Male	0	brain, supra-tentorial	12788	36.8	600809645	46982	2705	12576
										**71985**

Online Resource 8 (related to Figure 3): Overview of scRNA-seq Samples

Summary of all eight scRNA-seq samples.

Online Resource 9

Cluster	Total Number of Cells	Number of Smarcb1$^{+/+}$ Cells	Number of Smarcb1$^{1148del/1148del}$ Cells	Cell class	Dominating Cell Type	Cluster Annotation
**0**	7027	1765	5262	Neurons	Excitatory Neurons of the Forebrain 1 (upper layers)	ExcFore1 (LUpper)
**1**	4463	1870	2593	Neurons	Inhibitory Neurons of the Forebrain 1 (early, migrating)	InhFore1 (early, mig)
**2**	4280	1630	2650	Neurons	Inhibitory Neurons of the Forebrain 2 (incl. medium spiny neurons)	InhFore2 (incl. MSN)
**3**	4215	1237	2978	Neurons	Excitatory Neurons of the Forebrain 2 (early, migrating)	ExcFore2 (early, mig)
**4**	4144	1678	2466	Neurons	Excitatory Neurons of the Forebrain 3	ExcFore3
**5**	3872	1279	2593	Radial glia-like cells	Radial glia-like cells 1	Radial glia-like1
**6**	2592	1139	1453	Neurons	Inhibitory Neurons of the Midbrain 1	InhMid1
**7**	3446	1656	1790	Neurons	Excitatory Neurons of the Hindbrain 2 (granule neuron precursors)	ExcHind2
**8**	3319	1913	1406	Neurons	Neurons with Mixed Neurotransmitters 1	Mix1
**9**	3270	849	2421	Unassigned	Unassigned	Unassigned
**10**	2765	1328	1437	Neurons	Inhibitory Neurons of the Forebrain 3 (MGE-derived interneurons)	InhFore3 (MGE-Int.)
**11**	2418	871	1547	Oligodendrocyte lineage	Oligodendrocyte precursor cells	OPC
**12**	2533	1366	1167	Neurons	Inhibitory Neurons of the Midbrain 2	InhMid2
**13**	2163	628	1535	Neuroprogenitors	Neuroprogenitors 2 (cycling)	Neuroprogenitors2
**14**	2132	751	1381	Neuroprogenitors	Neuroblasts (early)	Neuroblasts (early)
**15**	2111	984	1127	Neurons	Excitatory Neurons of the Midbrain 1	ExcMid1
**16**	2078	841	1237	Neurons	Inhibitory Neurons of the Forebrain 4 (CGE-derived interneurons)	InhFore4 (CGE-Int.)
**17**	1935	849	1086	Neurons	Excitatory Neurons of the Hindbrain 1	ExcHind1
**18**	1860	747	1113	Neurons	Excitatory Neurons of the Hindbrain 3	ExcHind3
**19**	1514	377	1137	Neurons	Excitatory Neurons of the Forebrain 4 (hippocampal)	ExcFore4 (hpc)
**20**	1193	408	785	Neuroprogenitors	Neuroprogenitors 1 (cycling)	Neuroprogenitors1
**21**	1181	277	904	Radial glia-like cells	Radial glia-like cells 2	Radial glia-like2
**22**	1086	537	549	Neurons	Inhibitory Neurons of the Hindbrain 1	InhHind1

**23**	864	318	546	Radial glia-like cells	Radial glia-like cells 3	Radial glia-like3
**24**	857	328	529	Neurons	Neurons with Mixed Neurotransmitters 2	Mix2
**25**	713	230	483	Vascular cells	Vascular and Leptomeningeal cells	VLMC
**26**	394	161	233	Neurons	Inhibitory Neurons of the Hindbrain 2 (Purkinje cells)	InhHind2 (PC)
**27**	576	178	398	Immune cells	Immune Cells (Blood Monozytes, Mikroglia, Perivascular Macrophages, …)	Immune
**28**	546	116	430	Neurons	Excitatory Neurons of the Forebrain 5 (incl. deep layers)	ExcFore5 (LDeep)
**29**	174	83	91	Ciliated cells	Choroid Plexus Cells	Choroid plexus
**30**	254	116	138	Neurons	Excitatory Neurons of the Midbrain 2	ExcMid2
**31**	236	68	168	Oligodendrocyte lineage	Oligodendrocyte Precursor Cells (cycling)	OPC (cycling)
**32**	99	38	61	Vascular cells	Vascular Endothelial Cells	VEC
**33**	152	33	119	Ciliated cells	Ependymal Cells	Ependymal
**34**	1121	452	669	Neurons	Excitatory Neurons of the Midbrain 3	ExcMid3
**35**	180	68	112	Oligodendrocyte lineage	Committed Oligodendrocytes Precursors, Newly-Formed Oligodendrocytes and Myelin-Forming Oligodendrocytes	COP-NFOL-MFOL
**36**	222	61	161	Neurons	Cajal-Retzius Cells	Cajal-Retzius
**Total**	**71985**	**27230**	**44755**			

Online Resource 9 (related to Figure 3): Cell Numbers and Cell Type Annotation of scRNA-seq Clusters

Detailed cell numbers and cell type annotation for all clusters

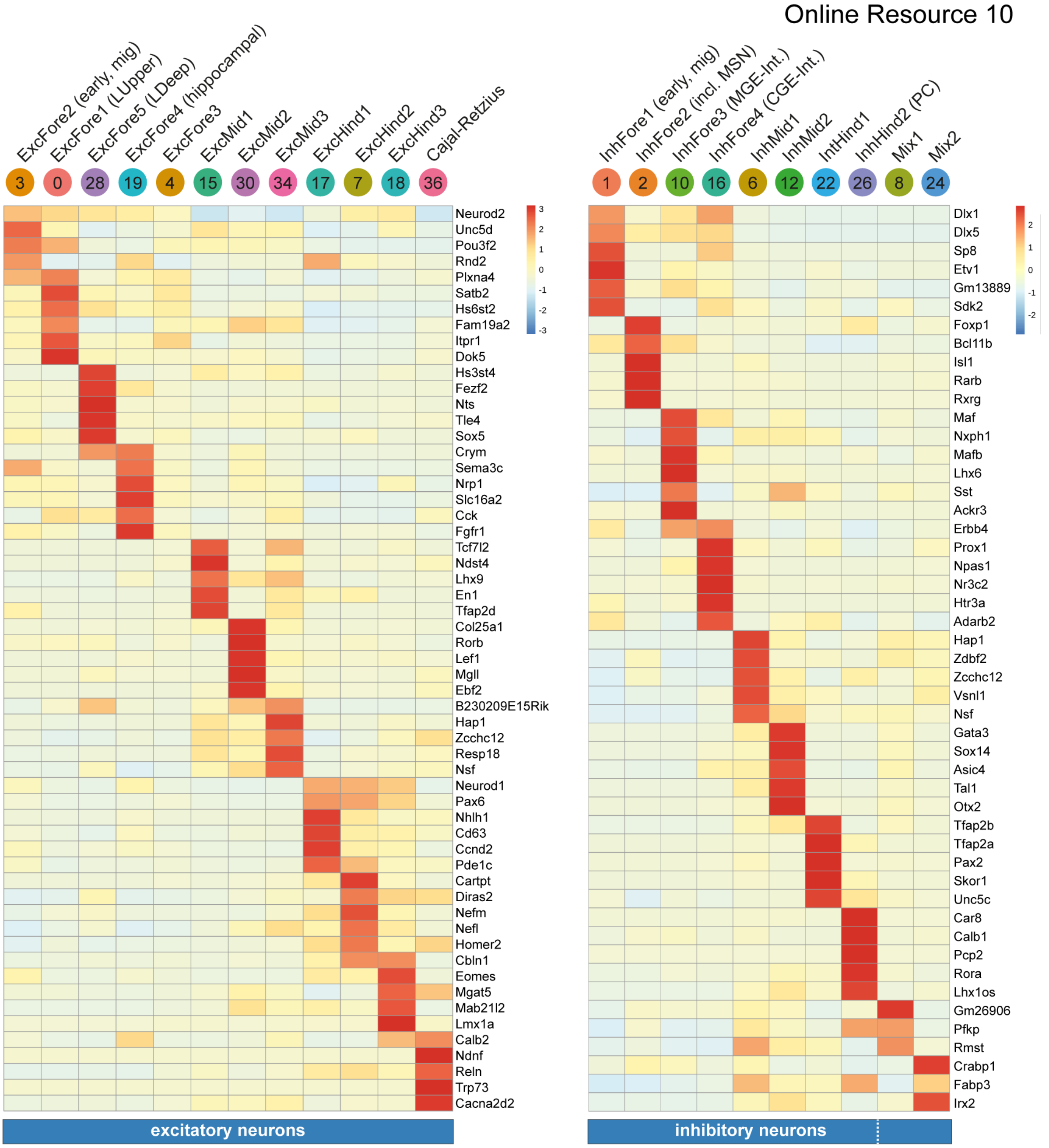

Online Resource 10 (related to Figure 3): Cell type markers of neuronal clusters

Heatmaps showing relative expression of specific marker genes for each cluster containing excitatory neurons (Exc), inhibitory neurons (Inh) or a mix of excitatory and inhibitory neurons (Mix). Fore, forebrain; Mid, midbrain; Hind; hindbrain; mig, migrating; LUpper, upper layers; LDeep, deep layers; MSN, medium spiny neurons; MGE, medium ganglionic eminence; CGE, caudal ganglionic eminence; Int, interneurons; PC, purkinje cells

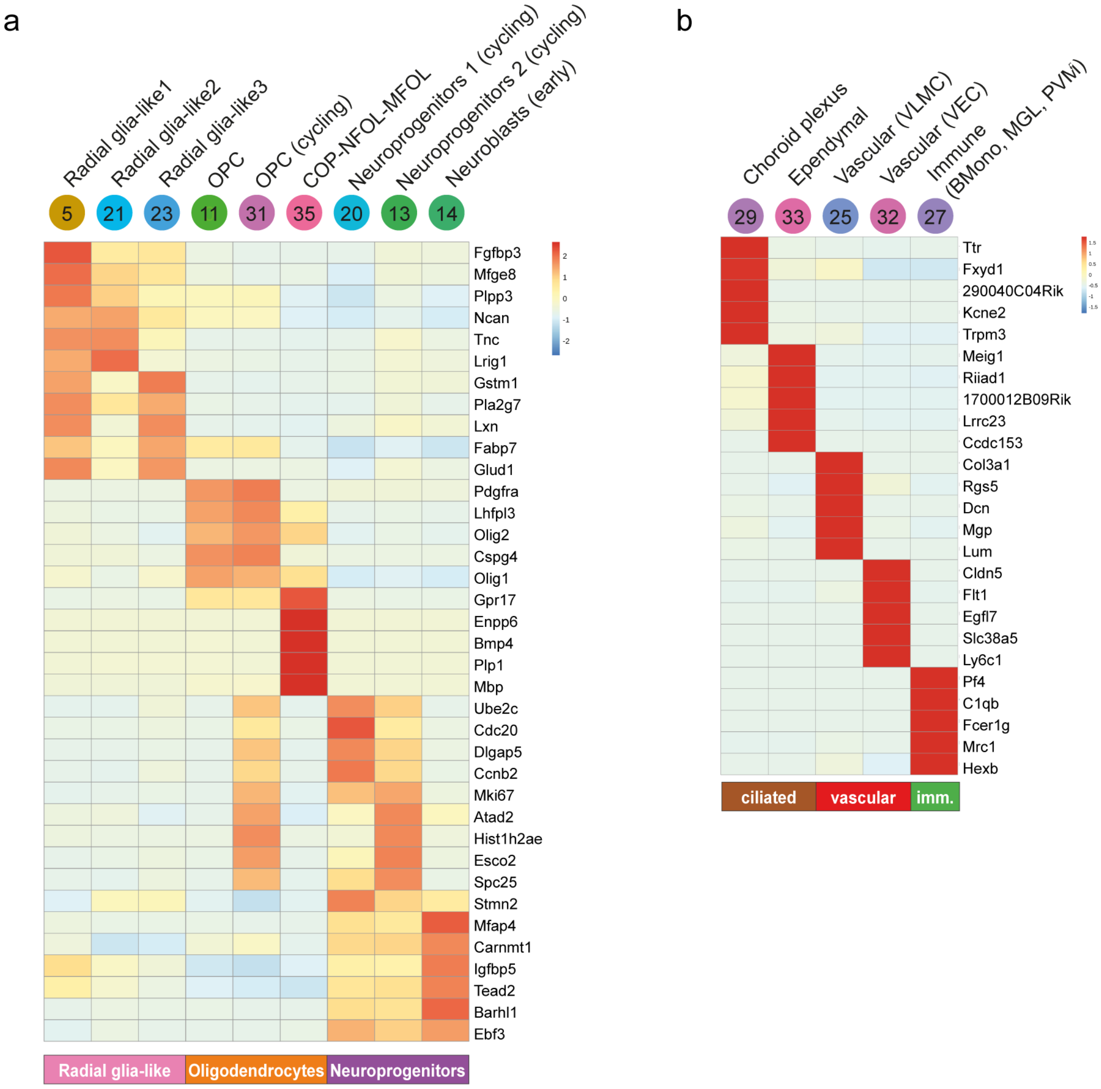

Online Resource 11 (related to Figure 3): Cell type markers of radial glia-like cells, cells of the oligodendrocyte lineage, neuroprogenitors, ciliated cells, vascular cells and immune cells

Heatmaps showing relative expression of specific marker genes for radial glia-like cells, cells of the oligodendrocyte lineage, neuroprogenitor cells and neuroblasts in **(a)**, and **(b)** for clusters containing choroid plexus, ependymal, vascular and immune (imm.) cells. OPC, oligodendrocyte precursor cells; COP, committed oligodendrocytes precursors; NFOL, newly-formed oligodendrocytes; MFOL, Myelin-forming oligodendrocytes; VLMC, vascular and meningeal cells; VEC, vascular endothelial cells; BMono, blood monocytes; MGL, Mikroglia; PVM, perivascular macrophages.

Statistical Analysis - Fig. 1d: MRI measurements of *Smarcb1*$^{+/+}$, *Smarcb1*$^{1148del/+}$ and *Smarcb1*$^{1148del/1148del}$ mice

Results of normality tests (Shapiro-Wilk test)

**Absolute Brain Volume**	*Smarcb1*$^{+/+}$:	$W(5) = 0.97, p = 0.878$
	Smarcb1$^{1148del/+}$:	$W(5) = 0.82, p = 0.123$
	Smarcb1$^{1148del/1148del}$:	$W(5) = 0.78, p = 0.060$
**Absolute Ventricle Volume**	*Smarcb1*$^{+/+}$:	$W(5) = 0.93, p = 0.621$
	Smarcb1$^{1148del/+}$:	$W(5) = 0.98, p = 0.919$
	Smarcb1$^{1148del/1148del}$:	$W(5) = 0.78, p = 0.058$
**Relative Ventricle Volume**	*Smarcb1*$^{+/+}$:	$W(5) = 0.92, p = 0.512$
	Smarcb1$^{1148del/+}$:	$W(5) = 0.98, p = 0.918$
	Smarcb1$^{1148del/1148del}$:	$W(5) = 0.78, p = 0.054$

Results of normality tests (Kolmogorov-Smirnov test with Lilliefors Significance Correction)

**Absolute Brain Volume**	*Smarcb1*$^{+/+}$:	$D(5) = 0.17, p = 0.200$
	Smarcb1$^{1148del/+}$:	$D(5) = 0.29, p = 0.196$
	Smarcb1$^{1148del/1148del}$:	$D(5) = 0.35, p = 0.043$
**Absolute Ventricle Volume**	*Smarcb1*$^{+/+}$:	$D(5) = 0.27, p = 0.200$
	Smarcb1$^{1148del/+}$:	$D(5) = 0.16, p = 0.200$
	Smarcb1$^{1148del/1148del}$:	$D(5) = 0.35, p = 0.042$
**Relative Ventricle Volume**	*Smarcb1*$^{+/+}$:	$D(5) = 0.27, p = 0.200$
	Smarcb1$^{1148del/+}$:	$D(5) = 0.15, p = 0.200$
	Smarcb1$^{1148del/1148del}$:	$D(5) = 0.35, p = 0.040$

For the measurements of *Smarcb1*$^{1148del/1148del}$ mice, the Shapiro-Wilk test and the Kolmogorov-Smirnov test show incongruent results. Since the Q-Q plot and the Kolmogorov-Smirnov test do not indicate a normal distribution, we assumed that the data is not normally distributed, and therefore performed a non-parametric test for all measurements.

Kruskal-Wallis test

Absolute Brain Volume test summary: $z = 2.06, p = 0.357$

pairwise comparisons:

Smarcb1$^{+/+}$ vs. *Smarcb1*$^{1148del/+}$: $z = 4.00, p = 0.157$

Smarcb1$^{+/+}$ vs. *Smarcb1*$^{1148del/1148del}$: $z = 2.60, p = 0.358$

Smarcb1$^{1148del/+}$ vs. *Smarcb1*$^{1148del/1148del}$: $z = 1.40, p = 0.621$

Absolute Ventricle Volume test summary: $z = 8.96, p = 0.011$

pairwise comparisons:

Smarcb1$^{+/+}$ vs. *Smarcb1*$^{1148del/+}$: $z = -1.60, p = 0.572$

Smarcb1$^{+/+}$ vs. *Smarcb1*$^{1148del/1148del}$: $z = -8.00, p = 0.005$

Smarcb1$^{1148del/+}$ vs. *Smarcb1*$^{1148del/1148del}$: $z = 6.40, p = 0.024$

Relative Ventricle Volume test summary: $z = 9.36, p = 0.009$

pairwise comparisons:

Smarcb1$^{+/+}$ vs. *Smarcb1*$^{1148del/+}$: $z = -2.40, p = 0.396$

Smarcb1$^{+/+}$ vs. *Smarcb1*$^{1148del/1148del}$: $z = -8.40, p = 0.003$

Smarcb1$^{1148del/+}$ vs. *Smarcb1*$^{1148del/1148del}$: $z = 6.00, p = 0.034$

Only the p-values for the absolute and relative ventricle volume of *Smarcb1*$^{+/+}$ vs. *Smarcb1*$^{1148del/1148del}$ meet the Bonferroni adjusted alpha level of 0.0167 per test (0.05/3).

Bonferroni corrected α-levels (k = 3)

$\alpha_{original} = 0.05$ $\alpha_{altered} = 0.05/3 = 0.0.0167$

$\alpha_{original} = 0.01$ $\alpha_{altered} = 0.01/3 = 0.0033$

$\alpha_{original} = 0.001$ $\alpha_{altered} = 0.001/3 = 0.0003$

	***Smarcb1*$^{+/+}$**					***Smarcb1*$^{1148del/+}$**					***Smarcb1*$^{1148del/1148del}$**				
	N	*N*(female)	*N*(male)	*M*	*SD*	*N*	*N*(female)	*N*(male)	*M*	*SD*	*N*	*N*(female)	*N*(male)	*M*	*SD*
**Absolute brain volume**	5	1	4	456.13 µL	11.83	5	1	4	440.81 µL	9.81	5	2	3	505.15 µL	104.26
**Absolute ventricle volume**	5	1	4	11.69 µL	1.68	5	1	4	13.63 µL	3.31	5	2	3	90.44 µL	102.08
**Relative ventricle volume**	5	1	4	0.0256	0.0034	5	1	4	0.0310	0.0081	5	2	3	0.1538	0.1532

Statistical Analysis - Fig. 4c and 4d: Expression of AP-1 transcription factor and neurite outgrowth-associated genes in *Smarcb1*$^{+/+}$ and *Smarcb1*$^{1148del/1148del}$ neurons

Results of normality tests (Anderson-Darling-Test)

Fos	*Smarcb1*$^{+/+}$:	$W(21151) = 1320.23, p < 0.0001$
	Smarcb1$^{1148del/1148del}$:	$W(31611) = 2631.63, p < 0.0001$
Fosb	*Smarcb1*$^{+/+}$:	$W(21151) = 3717.95, p < 0.0001$
	Smarcb1$^{1148del/1148del}$:	$W(31611) = 7412.19, p < 0.0001$
Jun	*Smarcb1*$^{+/+}$:	$W(21151) = 570.26, p < 0.0001$
	Smarcb1$^{1148del/1148del}$:	$W(31611) = 792.79, p < 0.0001$
Jund	*Smarcb1*$^{+/+}$:	$W(21151) = 729.90, p < 0.0001$
	Smarcb1$^{1148del/1148del}$:	$W(31611) = 614.29, p < 0.0001$
Stmn3	*Smarcb1*$^{+/+}$:	$W(21151) = 886.34, p < 0.0001$
	Smarcb1$^{1148del/1148del}$:	$W(31611) = 877.43, p < 0.0001$
Stmn2	*Smarcb1*$^{+/+}$:	$W(21151) = 852.87, p < 0.0001$
	Smarcb1$^{1148del/1148del}$:	$W(31611) = 1139.97, p < 0.0001$
Tubb2a	*Smarcb1*$^{+/+}$:	$W(21151) = 626.56, p < 0.0001$
	Smarcb1$^{1148del/1148del}$:	$W(31611) = 708.25, p < 0.0001$
Tubb3	*Smarcb1*$^{+/+}$:	$W(21151) = 1077.15, p < 0.0001$
	Smarcb1$^{1148del/1148del}$:	$W(31611) = 881.07, p < 0.0001$
Gap43	*Smarcb1*$^{+/+}$:	$W(21151) = 728.16, p < 0.0001$
	Smarcb1$^{1148del/1148del}$:	$W(31611) = 1028.55, p < 0.0001$
Basp1	*Smarcb1*$^{+/+}$:	$W(21151) = 1167.10, p < 0.0001$
	Smarcb1$^{1148del/1148del}$:	$W(31611) = 1589.49, p < 0.0001$

The Anderson-Darling test was used as an alternative to the Shapiro-Wilk test, since the latter is not applicable to very large data sets (n >> 5000, in the function "shapiro.test" of the R package "stats").

Results of normality tests (Kolmogorov-Smirnov test with Lilliefors Significance Correction)

Fos	*Smarcb1*$^{+/+}$:	$D(21151) = 0.27, p < 0.0001$
	Smarcb1$^{1148del/1148del}$:	$D(31611) = 0.31, p < 0.0001$
Fosb	*Smarcb1*$^{+/+}$:	$D(21151) = 0.43, p < 0.0001$
	Smarcb1$^{1148del/1148del}$:	$D(31611) = 0.47, p < 0.0001$
Jun	*Smarcb1*$^{+/+}$:	$D(21151) = 0.18, p < 0.0001$

	Smarcb1$^{1148del/1148del}$:	$D(31611) = 0.18, p < 0.0001$
Jund	*Smarcb1*$^{+/+}$:	$D(21151) = 0.13, p < 0.0001$
	Smarcb1$^{1148del/1148del}$:	$D(31611) = 0.12, p < 0.0001$
Stmn3	*Smarcb1*$^{+/+}$:	$D(21151) = 0.13, p < 0.0001$
	Smarcb1$^{1148del/1148del}$:	$D(31611) = 0.12, p < 0.0001$
Stmn2	*Smarcb1*$^{+/+}$:	$D(21151) = 0.12, p < 0.0001$
	Smarcb1$^{1148del/1148del}$:	$D(31611) = 0.12, p < 0.0001$
Tubb2a	*Smarcb1*$^{+/+}$:	$D(21151) = 0.14, p < 0.0001$
	Smarcb1$^{1148del/1148del}$:	$D(31611) = 0.15, p < 0.0001$
Tubb3	*Smarcb1*$^{+/+}$:	$D(21151) = 0.14, p < 0.0001$
	Smarcb1$^{1148del/1148del}$:	$D(31611) = 0.10, p < 0.0001$
Gap43	*Smarcb1*$^{+/+}$:	$D(21151) = 0.20, p < 0.0001$
	Smarcb1$^{1148del/1148del}$:	$D(31611) = 0.20, p < 0.0001$
Basp1	*Smarcb1*$^{+/+}$:	$D(21151) = 0.15, p < 0.0001$
	Smarcb1$^{1148del/1148del}$:	$D(31611) = 0.14, p < 0.0001$

The data showed non-normal distribution, we, therefore, performed a non-parametric test.

Independent Mann-Whitney U Test

Fos	$z = -29.16, p < 0.0001$
Fosb	$z = -30.16, p < 0.0001$
Jun	$z = -9.06, p < 0.0001$
Jund	$z = -40.19, p < 0.0001$
Stmn3	$z = -43.28, p < 0.0001$
Stmn2	$z = -29.87, p < 0.0001$
Tubb2a	$z = -40.71, p < 0.0001$
Tubb3	$z = -37.93 , p < 0.0001$
Gap43	$z = -15.76, p < 0.0001$
Basp1	$z = 97.75, p < 0.0001$

All p-values meet the Bonferroni adjusted alpha level of 0.0001 per test (0.001/10).

Bonferroni corrected α-levels (k = 10)

$\alpha_{original} = 0.05$	$\alpha_{altered} = 0.05/10 = 0.005$
$\alpha_{original} = 0.01$	$\alpha_{altered} = 0.01/12 = 0.0001$
$\alpha_{original} = 0.001$	$\alpha_{altered} = 0.001/12 = 0.0001$

Statistical Analysis – Online Resource 3d: Weight of *Smarcb1*$^{+/+}$ and *Smarcb1*$^{1148del/1148del}$ mice

Results of normality tests (Shapiro-Wilk test)

**30 days:**	*Smarcb1*$^{+/+}$:	$W(11) = 0.89, p = 0.148$
	Smarcb1$^{1148del/1148del}$:	$W(7) = 0.91, p = 0.391$
**35 days:**	*Smarcb1*$^{+/+}$:	$W(10) = 0.96, p = 0.787$
	Smarcb1$^{1148del/1148del}$:	$W(5) = 0.97, p = 0.851$
**40 days:**	*Smarcb1*$^{+/+}$:	$W(4) = 0.99, p = 0.972$
	Smarcb1$^{1148del/1148del}$:	$W(5) = 0.97, p = 0.899$

Results of normality tests (Kolmogorov-Smirnov test with Lilliefors Significance Correction)

**30 days:**	*Smarcb1*$^{+/+}$:	$D(11) = 0.23, p = 0.117$
	Smarcb1$^{1148del/1148del}$:	$D(7) = 0.17, p = 0.200$
**35 days:**	*Smarcb1*$^{+/+}$:	$D(10) = 0.16, p = 0.200$
	Smarcb1$^{1148del/1148del}$:	$D(5) = 0.23, p = 0.200$
**40 days:**	*Smarcb1*$^{+/+}$:	$D(4) = 0.15, p = .$
	Smarcb1$^{1148del/1148del}$:	$D(5) = 0.17, p = 0.200$

Results of Test for Homogeneity of Variances (Levene's Test)

**30 days:**	$F(1,16) = 1.18, p = 0.293$
**35 days:**	$F(1,13) = 1.27, p = 0.280$
**40 days:**	$F(1,7) = 0.04, p = 0.847$

The data showed no evidence of non-normality or inequality of variances, so we performed a parametric test.

Results of Tests for Equality of Means (Two-Sided Unpaired *t*-test)

**30 days:**	$t(16) = 5.60, p < 0.001$
**35 days:**	$t(13) = 6.50, p < 0.001$
**40 days:**	$t(7) = 3.13, p = 0.016$

	Smarcb1$^{+/+}$					***Smarcb1***$^{\mathbf{1148del/1148del}}$							
	N	*N*(female)	*N*(male)	*M* [g]	*SD*	*N*	*N*(female)	*N*(male)	*M* [g]	*SD*	*df*	*t*	*p*
**30 days**	11	4	7	16.00	1.73	7	3	4	10.43	2.51	16	5.60	< 0.001
**35 days**	10	7	3	18.87	2.16	5	3	2	11.82	1.49	13	6.50	< 0.001
**40 days**	4	0	4	21.00	2.58	5	3	2	15.80	2.39	7	3.13	0.016

References of Online Resources

Cheng SSW, Luk H, Mok MT, et al (2021) Genotype and phenotype in 18 Chinese patients with Coffin-Siris syndrome. Am J Med Genet A 185:2250–2261. https://doi.org/10.1002/ajmg.a.62187

Diets IJ, Prescott T, Champaigne NL, et al (2019) A recurrent de novo missense pathogenic variant in SMARCB1 causes severe intellectual disability and choroid plexus hyperplasia with resultant hydrocephalus. Genet Med 21:572–579. https://doi.org/10.1038/s41436-018-0079-4

Filatova A, Rey LK, Lechler MB, et al (2019) Mutations in SMARCB1 and in other Coffin–Siris syndrome genes lead to various brain midline defects. Nat Commun 10:2966. https://doi.org/10.1038/s41467-019-10849-y

Gossai N, Biegel JA, Messiaen L, et al (2015) Report of a patient with a constitutional missense mutation in SMARCB1 , Coffin-Siris phenotype, and schwannomatosis. Am J Med Genet Part A 167A:3186–3191. https://doi.org/10.1002/ajmg.a.37356

Kleefstra T, Kramer JM, Neveling K, et al (2012) Disruption of an EHMT1-Associated Chromatin-Modification Module Causes Intellectual Disability. Am J Hum Genet 91:73–82. https://doi.org/10.1016/j.ajhg.2012.05.003

Lee Y, Choi Y, Seo GH, et al (2021) Phenotypic and molecular spectra of patients with switch/sucrose nonfermenting complex-related intellectual disability disorders in Korea. BMC Med Genomics 14:1–10. https://doi.org/10.1186/s12920-021-01104-9

Santen GWE, Aten E, Vulto-van Silfhout AT, et al (2013) Coffin-Siris Syndrome and the BAF Complex: Genotype-Phenotype Study in 63 Patients. Hum Mutat 34:1519–1528. https://doi.org/10.1002/humu.22394

Sekiguchi F, Tsurusaki Y, Okamoto N, et al (2019) Genetic abnormalities in a large cohort of Coffin–Siris syndrome patients. J Hum Genet 64:1173–1186. https://doi.org/10.1038/s10038-019-0667-4

Tate JG, Bamford S, Jubb HC, et al (2019) COSMIC: the Catalogue Of Somatic Mutations In Cancer. Nucleic Acids Res 47:D941–D947. https://doi.org/10.1093/nar/gky1015

Tsurusaki Y, Okamoto N, Ohashi H, et al (2014) Coffin-Siris syndrome is a SWI/SNF complex disorder. Clin Genet 85:548–554. https://doi.org/10.1111/cge.12225

Tsurusaki Y, Okamoto N, Ohashi H, et al (2012) Mutations affecting components of the SWI/SNF complex cause Coffin-Siris syndrome. Nat Genet 44:376–378. https://doi.org/10.1038/ng.2219

Wieczorek D, Bögershausen N, Beleggia F, et al (2013) A comprehensive molecular study on Coffin–Siris and Nicolaides–Baraitser syndromes identifies a broad molecular and clinical spectrum converging on altered chromatin remodeling. Hum Mol Genet 22:5121–5135. https://doi.org/10.1093/hmg/ddt366
